# ﻿A survey of scale insects (Hemiptera, Coccoidea) on avocados, olives, and grapes in the Peruvian region of Arequipa

**DOI:** 10.3897/zookeys.1257.163722

**Published:** 2025-10-28

**Authors:** Maholy Beltran-Moreno, Anali Llacctas, Javier Huanca, Gregory Evans

**Affiliations:** 1 Entomology Laboratory, Faculty of Biological Sciences, Universidad Nacional de San Agustin de Arequipa, Arequipa, Peru Universidad Nacional de San Agustin de Arequipa Arequipa Peru; 2 APHIS, United States Department of Agriculture, Beltsville, Maryland, 20705, USA United States Department of Agriculture Beltsville United States of America

**Keywords:** Coccomorpha, *Olea europaea*, *Persea americana*, phytophagous, *Vitis vinifera*

## Abstract

Scale insects (Hemiptera, Coccoidea) are among the most economically important plant pests known throughout the world. They are found frequently on the leaves, stems, roots, and fruit, and are of great concern to importers of these plant products due to their great potential to become invasive species in their new environment when separated from their natural enemies. In Peru, knowledge of scale insects is limited to a few economically important species primarily because local, regional, or national surveys have not been conducted. This study aimed to document and update knowledge of scale insect diversity on avocado, olive, and grape plantations in the Arequipa region of Peru. Samples were collected from fruit orchards in June 2019, February–March 2020, August 2021, and July 2022. Sampling was performed in 35 localities across eight provinces in the Arequipa region where crops are grown for commercial purposes. Leaves, branches, and fruit of each of these three crops at each locality were randomly sampled, and inspected for the presence of scale insects. A total of 13,991 scale insect specimens were collected from 668 samples. Diversity indices were used to assess the structure of the scale insect communities across the three crops. Five families, 22 genera, and 33 species were identified. Species in the family Diaspididae were the most abundant, followed by Coccidae, Pseudococcidae, Ortheziidae and Eriococcidae, respectively. Twenty-eight species of scale insects are reported for the first time in the Arequipa region, nine of which are new records for Peru. The highest species richness was found on grapes, followed by avocados, and the lowest richness was found in olives. A dichotomous morphological key to the species of scale insects reported in this study is provided.

## ﻿Introduction

Scale insects constitute a diverse group of plant-feeding insects found across all of the major terrestrial biogeographic regions of the world, except Antarctica. They constitute one of the largest groups of insects associated with fruit trees, with 133 species reported on *Persea americana* Mill. (Lauraceae), 125 species on *Vitis vinifera* L. (Vitaceae), and 108 species on *Olea europaea* L. (Oleaceae); this information was extracted from the scale insect database, ScaleNet (Garcia Morales et al. 2016), a website that includes information on the taxonomy, distribution, and ecology of species of scale insects known worldwide based on published records.

Several species of scale insects belonging to different families have become invasive pests that pose a threat to agroecosystems, and impact the trade of plant products worldwide (Miller et al. 2005). These include mealybugs, which are important economic pests such as *Pseudococcus viburni* (Signoret, 1875), *Pseudococcus longispinus* (Targion-Tozzetti, 1867), *Planococcus ficus* (Signoret, 1875), and *Planococcus citri* (Risso, 1813), which affect a wide variety of crops in Peru (Salazar 1972). Armored scale insects are considered among the most invasive insects in the world (Miller et al. 2005). Of particular concern are species that have been reported on grapevines in Brazil, such as *Melanaspis arnaldoi* (Costa Lima, 1924), which infests tree trunks, and *Aonidiella orientalis* (Newstead, 1894) collected on grape leaves, and fruit (Costa Lima et al. 2024). Soft scale insects have likewise proven highly destructive to cultivated plants. Examples include *Saissetia oleae* (Olivier, 1791) on olive trees in the USA, Israel, Australia, and Japan, *Filippia follicularis* (Targioni Tozzetti, 1867) on Mediterranean olive trees, and *Parthenolecanium corni* (Bouché, 1844) on grapevines in the USA and Europe (Gill and Kosztarab 1997).

Previous studies have documented the taxonomic diversity of the fauna of scale insects in Peru, particularly in relation to economically important crops. Thirteen species have been reported on olives (Beingolea 1965, 1969b; Gonzalez 1966; Beingolea and Salazar 1970; Salazar 1972; Aguilar et al. 1980; Marin-Loayza and Cisneros 1982), with the following four species found on olives in the Arequipa region: *Hemiberlesia lataniae* (Signoret,1859), *Saissetia oleae*, *Saissetia coffeae* (Walker, 1852), and *Praelongorthezia olivicola* (Beingolea, 1965). Seven scale insect species have been reported on avocados in Peru (Bartra 1976; Marin-Loayza and Cisneros 1979, 1982; Garcia-Morales et al. 2016; Collantes and Rodriguez 2022); however, no records were reported from the Arequipa region. Grapes have been reported to host five scale insect species in Peru (Gonzalez 1966; Salazar 1972; Marin-Loayza and Cisneros 1979; Granara de Willink and Diaz 2007; Garcia-Morales et al. 2016), with *H. lataniae*, and *Ovaticoccus peruvianus* (Granara de Willink & Diaz, 2007) found in Arequipa. Notably, two species were originally described from specimens collected in Peru: *P. olivicola*, associated with olives (Beingolea 1965), and *O. peruvianus*, associated with grapes (Granara de Willink and Diaz 2007).

Correct pest identification practices are crucial for detecting invasive pests on plant products in international commerce, and for developing strategies for effective integrated pest management programs (Costa Lima et al. 2024). Documenting the presence, distribution, biology, natural enemies, and host associations of pest species provides a valuable baseline for identifying which species are already established in a region or country (Kakoti et al. 2023).

In the Arequipa region, the area devoted to fruit growing is increasing every year, reflecting the region’s growing role in agricultural production. Peru is among the countries actively involved in the export and import of unprocessed agricultural goods (MIDAGRI 2025).

This paper provides an updated list of scale insects associated with avocados, grapes and olives of the Arequipa region during the period of 2019 to 2022. In addition, we present data on abundance, and distribution of scale insect species in the Arequipa region, and a dichotomous morphological key of species.

## ﻿Materials and methods

### ﻿Sampling

The study was carried out between June 2019, and July 2022 in eight provinces of the Arequipa region: Arequipa, Camana, Caraveli, Castilla, Caylloma, Condesuyos, Islay, and La Union. (Suppl. material 1). Agricultural farms were selected in three localities in each of the eight provinces. Sampling was conducted on three plots on each farm where 20 plants each of *Olea europaea* L. (Oleaceae), *Persea americana* Mill. (Lauraceae), and *Vitis vinifera* L. (Vitaceae), were selected. Leaves, branches, and fruit were examined for the presence of scale insects. Samples infested with scale insects were placed in polyethylene zip-lock bags, which were stored in hermetic boxes containing frozen gel blocks before being transported to the Entomology Laboratory of the Faculty of Biological Sciences of the Universidad Nacional de San Agustin de Arequipa. A total of 668 samples were collected; of these, 268 are from avocados, 228 from grapes, and 172 from olives.

### ﻿Identification of scale insects

Scale insects were extracted, and preserved in 20 ml glass vials containing 75% Ethanol, and sealed with hermetic lids. The specimens were mounted following methods described by several authors: for CoccidaeWilliams (1972); for DiaspididaeMcKenzie (1956), Henderson (2011), Schneider et al. (2020); for EriococcidaeMcKenzie (1967); for OrtheziidaePadilla et al. (2016); and for PseudococcidaeWilliams and Watson (1988).

Specimens were examined using a phase contrast microscope (Leica model DM 2500), and identified using specialized literature: for CoccidaeTakahashi (1955), Williams (1972), Pellizzari and Camporese (1994), Marin-Loayza and Cisneros (1995), Hodges (2002), Peronti et al. (2008), Fetykó and Kozár (2012), and Miller et al. (2014); for DiaspididaeMcKenzie (1956), McDaniel (1974), Miller and Davidson (2005), Dooley and Dones (2008), Wolff (2008), Evans et al. (2009), Miller et al. (2014), Liu and Feng (2018), Normark et al. (2019), Schneider et al. (2019), and Schneider et al. (2020); for EriococcidaeGranara de Willink and Diaz (2007); for OrtheziidaeBeingolea (1965); for PseudococcidaeWilliams (1962), Cox (1981, 1989), Williams and Granara (1992), Moghaddam (2013), Miller et al. (2014), Soysouvanh et al. (2015), and Zhang and Deng (2023).

Voucher specimens were deposited in the reference collection of the Entomology and Acarology Collection at the Universidad Nacional de San Agustin (**EACSAU**) in Arequipa, Peru.

### ﻿Data analysis

Species richness, and its similarity between hosts were analyzed. Alpha diversity (α), the observed richness (number of taxa) or evenness (relative abundances of those taxa) of an average sample within a habitat type, was measured using the Shannon - Wiener, Simpson, Margalef, Dominance, Evenness, and Berger-Parker Indexes. Beta diversity (β), the variability in community composition (the identity of taxa observed) among samples within a habitat, was measured using the Sorensen, Jaccard, and Morisita similarity coefficients. The statistical analysis was done using Paleontological Statistics Software v. 4.17c (Hammer et al. 2001).

## ﻿Results

### ﻿Species composition and relative abundance

**Figure 1.**
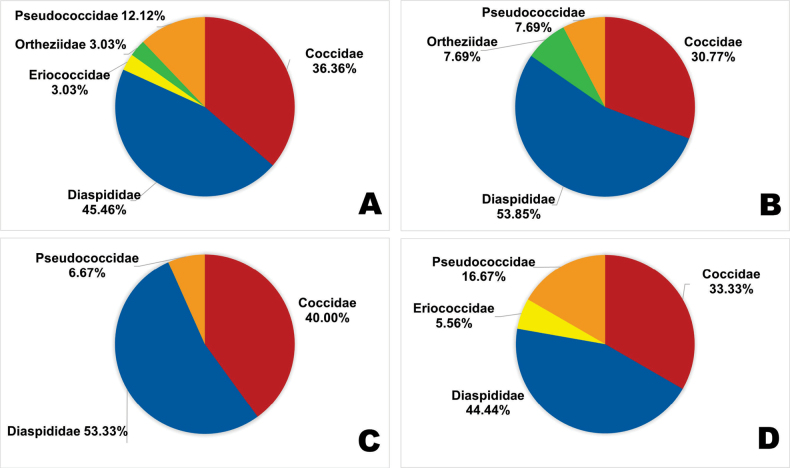
Proportions of scale insect community composition in (A) Arequipa region (B) *Olea europaea* (C), *Persea americana* (D) *Vitis vinifera*.

A total of 13,991 scale insect specimens were collected in the study. Of these, 8,062 were collected from avocados, 3,932 from olives, and 1,997 from grapes (Tables 1, 3). The two most abundant scale insect families in the region were Diaspididae (45.46%), and Coccidae (36.36%). In contrast, three families Pseudococcidae (12.12%), Ortheziidae (3.03%), and Eriococcidae (3.03%) were the least abundant (Fig. 1A). Similar composition and abundance of Diaspididae, Coccidae, and Pseudococcidae were found across the three surveyed crops (Fig. 1B–D), whereas Eriococcidae, was represented only on grapes (Fig. 1B), and Ortheziidae was found only on olives (Fig. 1D).

**Table 1.** Diversity and abundance of scale insects on *Olea europaea* L., *Persea americana* Mill., and *Vitis vinifera* L. in the Arequipa region, Peru.

Family	Genera	Total Species	(%)	Abundance
Coccidae	7	12	36.36	2523
Diaspididae	11	15	45.46	9762
Eriococcidae	1	1	3.03	332
Ortheziidae	1	1	3.03	666
Pseudococcidae	2	4	12.12	708
Total	22	33	100	13991

Several scale insects showed crop specificity, where some species were observed associated to a single host, six on avocado: *Protopulvinaria pyriformis* (Cockerell, 1894), *Saissetia neglecta* De Lotto, 1969, *Melanaspis squamea* Ferris, 1943, *Pseudoparlatoria parlatorioides* (Comstock, 1883), *Selenaspidus articulatus* (Morgan, 1889), *Planococcus citri*; eight on olives: *Kilifia acuminata* (Signoret, 1873), *Pulvinaria psidii* Maskell, 1893, *Saissetia coffeae*, *Saissetia oleae* (Olivier, 1791), *Furchadaspis zamiae* (Morgan, 1890), *Hemiberlesia palmae* (Cockerell, 1893), *Pinnaspis strachani* (Cooley, 1899), *Praelongorthezia olivicola*, and nine on grapes: *Ceroplastes floridensis* Comstock, 1881, *Ceroplastes sinensis* Del Guercio, 1900, *Coccus longulus* (Douglas, 1887), *Hemiberlesia rapax* (Comstock, 1881), *Oceanaspidiotus spinosus* (Comstock, 1883), *Pseudischnaspis bowreyi* (Cockerell, 1893), *O. peruvianus*, *Planococcus ficus*, *Pseudococcus viburni*; whereas others species were found to overlap among crops. Specifically, four species were shared across all three hosts: *Aspidiotus nerii* Bouche, 1833, *Hemiberlesia cyanophylli* (Signoret, 1869), *H. lataniae*, *Pseudococcus longispinus*. One species, *Fiorinia fioriniae* (Targioni Tozzetti, 1867), was shared between avocados and olives, while five species were found on both avocados and grapes: *Ceroplastes rusci* (L., 1758), *Coccus hesperidum* L., 1758, *Parasaissetia nigra* (Nietner, 1861), *Chrysomphalus dictyospermi* (Morgan, 1889), and *Pinnaspis aspidistrae* (Signoret, 1869). Notably, no species were found to be shared between olives and grapes, suggesting a more distinct scale insect community between these two crops (Table 2)

**Table 2.** Distribution of scale insects by host plant, and province in the Arequipa region, Peru.

Family/Species	Provinces							
	ARE	CAM	CAR	CAS	CAY	CON	ISL	LAU
** Coccidae **								
*Ceroplastes floridensis* Comstock, 1881	–	–	–	V	–	–	–	–
*Ceroplastes rusci* (Linnaeus, 1758)	–	–	–	P, V	–	V	–	–
*Ceroplastes sinensis* Del Guercio, 1900	–	V	–	–	–	–	–	–
*Coccus hesperidum* Linnaeus, 1758	P, V	P, V	P, V	P, V	P	P, V	–	P
*Coccus longulus* (Douglas, 1887)	–	V	V	V	–	V	–	–
*Kilifia acuminata* (Signoret, 1873)	–	–	O	O	–	O	O	–
*Parasaissetia nigra* (Nietner, 1861)	P, V	P, V	V	P, V	–	V	–	–
*Protopulvinaria pyriformis* (Cockerell, 1894)	–	P	–	P	–	–	–	–
*Pulvinaria psidii* Maskell, 1893	O	–	–	–	–	–	–	–
*Saissetia coffeae* (Walker, 1852)	–	O	O	O	O	O	O	–
*Saissetia neglecta* De Lotto, 1969	–	–	P	–	–	–	–	–
*Saissetia oleae* (Olivier, 1791)	–	O	O	O	O	–	O	–
** Diaspididae **								
*Aspidiotus nerii* Bouche, 1833	P, O, V	O, V	P, O, V	P, O	O	P, O, V	O	P, V
*Chrysomphalus dictyospermi* (Morgan, 1889)	P, V	P, V	P	P, V	–	P, V	–	–
*Fiorinia fioriniae* (Targioni Tozzetti, 1867)	–	P	O	P	–	–	O	–
*Furchadaspis zamiae* (Morgan, 1890)	–	–	O	–	–	–	–	–
*Hemiberlesia cyanophylli* (Signoret, 1869)	P, V	P, O, V	P, O, V	P, O	P, V	P	–	–
*Hemiberlesia lataniae* (Signoret, 1869)	P, V	P, O, V	P, O, V	P, O, V	P	P, O, V	O	P, V
*Hemiberlesia palmae* (Cockerell, 1893)	–	O	O	O	O	–	O	–
*Hemiberlesia rapax* (Comstock, 1881)	V	V	V	V	–	V	–	–
*Melanaspis squamea* Ferris, 1943	–	–	–	–	–	P	–	–
*Oceanaspidiotus spinosus* (Comstock, 1883)	–	–	V	V	–	V	–	–
*Pinnaspis aspidistrae* (Signoret, 1869)	–	P, V	–	–	–	–	–	–
*Pinnaspis strachani* (Cooley, 1899)	–	O	–	–	–	–	–	–
*Pseudischnaspis bowreyi* (Cockerell, 1893)	–	V	–	–	–	–	–	–
*Pseudoparlatoria parlatorioides* (Comstock, 1883)	P	P	P	P	–	P	–	P
*Selenaspidus articulatus* (Morgan, 1889)	–	–	–	P	–	–	–	–
** Eriococcidae **								
*Ovaticoccus peruvianus* (Granara de Willink and Diaz 2007)	V	–	–	V	–	V	–	–
** Ortheziidae **								
*Praelongorthezia olivicola* (Beingolea 1965)	–	O	O	–	–	–	O	–
** Pseudococcidae **								
*Planococcus citri* (Risso, 1813)	–	–	–	–	–	P	–	–
*Planococcus ficus* (Signoret, 1875)	V	–	–	V	V	–	–	–
*Pseudococcus longispinus* (Targioni Tozzetti, 1867)	–	P, O, V	O	P, O, V	–	V	O	–
*Pseudococcus viburni* (Signoret, 1875)	–	–	–	–	V	–	–	–

Host: *Olea europaea* (O); *Persea americana* (P); *Vitis vinifera* (V); Absent (-). Provinces: Arequipa (ARE); Camana (CAM); Caraveli (CAR); Castilla (CAS); Caylloma (CAY); Condesuyos (CON); Islay (ISL); La Union (LAU).

### ﻿Species richness and diversity

The highest species diversity was recorded on *V. vinifera*, with 18 species identified, followed by *P. americana* with 16 species, and *O. europaea* with 13 species (Table 3). This study represents the first comprehensive inventory of insects associated with these three crops in Arequipa, Peru.

**Table 3.** Species diversity, and species richness indices calculated for scale insects associated with three cultivated plants in Arequipa region.

Diversity Indexes	Hosts		
	Persea americana	Olea europaea	Vitis vinifera
Taxa	16	13	18
Individuals	8062	3932	1997
Shannon H	2.21	2.11	2.44
Margalef	1.67	1.45	2.24
Simpson 1-D	0.86	0.83	0.89
Evenness e^H/S	0.57	0.63	0.64
Berger-Parker	0.27	0.32	0.17

The community indices, specifically Margalef (d), ShannonWiener (H’), Simpson (1-D), and evenness were calculated in each agroecosystem (Table 3). The results show that Margalef, Shannon-Wiener and Simpson indices were higher in *V. vinifera* (d = 2.24, H’ = 2.44, 1-D = 0.89) than in the *P. americana* (d = 1.67, H’ = 2.21, 1-D = 0.86), and *O. europaea* (d = 1.45, H’ = 2.11, 1-D = 0.83) (Table 3). The evenness was similar in *V. vinifera* (0.64), *P. americana* (0.57), and *O. europaea* (0.63) (Table 3). The Berger-Parker dominance index showed a lower predominance of the most abundant species values in *V. vinifera* (0.17) than in the *P. americana* (0.27), and *O. europaea* (0.32) (Table 3).

The Margalef index (d), which measures species richness, showed that *V. vinifera* had the highest species richness, followed by *P. americana*, and *O. europaea*, indicating greater insect diversity associated with *V. vinifera*. The Shannon-Wiener, and Simpson diversity indices indicate that *V. vinifera* supports the most diverse scale insect community among the three crops analyzed. Where *V. vinifera* recorded the highest values for both indices, reflecting both high species richness and evenness. In comparison, *P. americana* showed moderate diversity, while *O. europaea* had the lowest diversity values. These findings suggest that *V. vinifera* provides a more favorable or complex habitat for scale insects, promoting a more balanced and varied community structure than the other two host plants.

Evenness values were moderately high and relatively similar across the three crops, indicating a fairly balanced distribution of individuals among species. Although, *P. americana* showed slightly lower evenness, no crop exhibited strong dominance by a single species, suggesting a moderately uniform scale insect community structure across the agroecosystems. The Berger-Parker dominance index showed lower values in the three surveyed crops. These results indicate a low predominance of the most abundant species across all three hosts, suggesting that no single species dominated the scale insect communities. This supports the overall balance in species distribution within each agroecosystem.

From an ecological perspective, the high diversity of scale insect species, particularly in *V. vinifera*, may indicate a stable agroecosystem but also presents challenges for pest management. The coexistence of multiple species increases the risk of certain ones becoming dominant pests under favorable conditions.

The similarity indices of Jaccard’s (Sj), Sorensen’s (Ss), and Morisita’s coefficient (Mo), revealed notable dissimilarity among the scale insect communities associated with the three crops. The lowest similarity was observed between *O. europaea* and *V. vinifera* (Sj = 0.15; Ss = 0.26; Mo = 0.12), indicating highly distinct communities. *P. americana* and *O. europaea* also showed low similarity (Sj = 0.21; Ss = 0.34; Mo = 0.23), while the highest, though still moderate, similarity was found between *P. americana* and *V. vinifera* (Sj = 0.36; Ss = 0.53; Mo = 0.30; Table 4). These results suggest that each crop hosts a largely unique set of scale insect species.

**Table 4.** Sorensen, Jaccard, and Morisita similarity coefficient between pair hosts about Coccoidea species present in Arequipa region. S_j_: Jaccard index; S_s_: Sorensen index; Mo: Morisita Index.

Hosts	Persea americana			Olea europaea			Vitis vinifera		
	S_s_	S_j_	Mo	S_s_	S_j_	Mo	S_s_	S_j_	Mo
*Persea americana*	1.00	1.00	1.00	0.34	0.21	0.23	0.53	0.36	0.30
*Olea europaea*	0.34	0.21	0.23	1.00	1.00	1.00	0.26	0.15	0.12
*Vitis vinifera*	0.53	0.36	0.30	0.26	0.15	0.12	1.00	1.00	1.00

### ﻿Scale insect distribution and their plant host

We present an updated and comprehensive list of scale insect species associated with olives (*Olea europaea*), avocados (*Persea americana*), and grapes (*Vitis vinifera*) in the Arequipa region of Peru. This list includes new distribution records as well as previously cited occurrences of several species in the country, thereby contributing valuable data to the national inventory of scale insects. Additionally, information on the presence of these species in Peru is provided (Table 5).

**Table 5.** Scale insect records and their plant hosts: avocados (*Persea americana*), grapes (*Vitis vinifera*) and olives (*Olea europaea*) from the Arequipa region, and Peru. Record citations of the species are represented by an “x,” and new host and distribution records are indicated by the word “new” under the columns for each crop, Arequipa, and Peru.

Family/ Species	Peru	Arequipa	Avocados	Grapes	Olives	Record citation
** Coccidae **						
*Ceroplastes floridensis* Comstock, 1881	x	new		new		Marin-Loayza and Cisneros (1996); Couturier et al. (1997)
*Ceroplastes rusci* (Linnaeus, 1758)	x	new	new	new		Marin-Loayza and Cisneros (1995)
*Ceroplastes sinensis* Del Guercio, 1900	new	new		new		
*Coccus hesperidum* Linnaeus, 1758	x	new	new	new		Beingolea (1967); Bederski (1969)
*Coccus longulus* (Douglas, 1887)	new	new		new		
*Kilifia acuminata* (Signoret, 1873)	new	new			new	
*Parasaissetia nigra* (Nietner, 1861)	x	new	x	x		Marin-Loayza and Cisneros (1979)
*Protopulvinaria pyriformis* (Cockerell, 1894)	x	new	new			Couturier et al. (1997)
*Pulvinaria psidii* Maskell, 1893	new	new			new	
*Saissetia coffeae* (Walker, 1852)	x	x			x	Beingolea (1967); Beingolea and Salazar (1970); Aguilar et al. (1980)
*Saissetia neglecta* De Lotto, 1969	new	new	new			
*Saissetia oleae* (Olivier, 1791)	x	x			x	Gonzalez (1966); Beingolea (1967); Beingolea and Salazar (1970); Aguilar et al. (1980)
** Diaspididae **						
*Aspidiotus nerii* Bouche, 1833	x	new	x	new	x	Beingolea and Salazar (1970); Bartra (1976)
*Chrysomphalus dictyospermi* (Morgan, 1889)	x	new	x	new		Beingolea (1967); Beingolea (1969a); Nuñez (2008)
*Fiorinia fioriniae* (Targioni Tozzetti, 1867)	x	new	x		new	Collantes and Rodriguez (2022)
*Furchadaspis zamiae* (Morgan, 1890)	new	new			new	
*Hemiberlesia cyanophylli* (Signoret, 1869)	x	new	x	new	x	Beingolea and Salazar (1970); Vasquez et al. (2002); Nuñez (2008)
*Hemiberlesia lataniae* (Signoret, 1869)	x	x	x	x	x	Gonzalez (1966); Beingolea and Salazar (1970); Nuñez (2008)
*Hemiberlesia palmae* (Cockerell, 1893)	x	new			new	Vasquez et al. (2002)
*Hemiberlesia rapax* (Comstock, 1881)	x	new		new	x	Beingolea and Salazar (1970); Nakahara (1982)
*Melanaspis squamea* Ferris, 1943	new	new	new			
*Oceanaspidiotus spinosus* (Comstock, 1883)	x	new		new		Nakahara (1982)
*Pinnaspis aspidistrae* (Signoret, 1869)	x	new	x	new		Marin-Loayza and Cisneros (1982); Collantes and Rodriguez (2022)
*Pinnaspis strachani* (Cooley, 1899)	x	new			x	Marin-Loayza and Cisneros (1982)
*Pseudischnaspis bowreyi* (Cockerell, 1893)	x	new		new	x	Beingolea and Salazar (1970); Miller et al. (1984)
*Pseudoparlatoria parlatorioides* (Comstock, 1883)	new	new	new			
*Selenaspidus articulatus* (Morgan, 1889)	x	new	new		x	Beingolea (1967); Beingolea (1969b); Beingolea and Salazar (1970)
** Eriococcidae **						
*Ovaticoccus peruvianus* (Granara de Willink and Diaz 2007)	x	x		x		Granara de Willink and Diaz (2007)
** Ortheziidae **						
*Praelongorthezia olivicola* (Beingolea 1965)	x	x			x	Beingolea (1965); Beingolea and Salazar (1970)
** Pseudococcidae **						
*Planococcus citri* (Risso, 1813)	x	new	x	x		Beingolea (1967); Bederski (1969); Salazar (1972); Ben-Dov (1994); De la Cruz (1996)
*Planococcus ficus* (Signoret, 1875)	new	new		new		
*Pseudococcus longispinus* (Targioni Tozzetti, 1867)	x	new	x	x	x	Salazar (1972); Ben-Dov (1994)
*Pseudococcus viburni* (Signoret, 1875)	x	new		x		Salazar (1972); Ben-Dov (1994)

Nine species of scale insects are new records for Peru: *S. neglecta*, *M. squamea*, and *P. parlatorioides* on avocados; *K. acuminata*, *Pulvinaria psidii*, and *Furchadaspis zamiae* on olives; and *Ceroplastes sinensis*, *Coccus longulus*, and *Planococcus ficus* on grapes (Table 5).

### ﻿Material studied

#### ﻿Coccidae

##### ﻿ Ceroplastes floridensis

Taxon classificationAnimaliaHemipteraCoccidae

Comstock, 1881

133745B1-8FA7-5EF3-972C-18B281186ED8

Suppl. material 1: fig. S2A, B

###### Distribution.

Peru: Castilla.

###### Examined material.

• 1 adult female, ex *Vitis vinifera*, Huancarqui (Castilla), 12/07/2019, A Llacctas.

###### Remarks.

This is the first record of this species found on grapes in Arequipa. Couturier et al. (1997) reported it on Myrtaceae fruit trees in Pucallpa, Peru; Marin-Loayza and Cisneros (1996) reported it on orange trees in Cañete Lima, Peru. The species has been found on avocados in Cuba (Ballou 1926), and Israel (Ben-Dov 1970a), and the United States Virgin Islands (Nakahara 1983).

##### ﻿ Ceroplastes rusci

Taxon classificationAnimaliaHemipteraCoccidae

(Linnaeus, 1758)

F6A3BC95-EAC2-59AF-923E-83133FB342B3

Suppl. material 1: fig. S2C, D

###### Distribution.

Peru: Castilla, Condesuyos.

###### Examined material.

• 19 adult females, ex *Persea americana*, Huancarqui (Castilla), 21/08/2021, J Jihuallanca; • 2 adult females, ex *Vitis vinifera*, La Barrera-Yanaquihua (Condesuyos), 13/03/2020, A Llacctas; • 2 adult females, ex *Vitis vinifera*, Luchea-Aplao (Castilla), 14/03/2020, A Llacctas; • 1 adult female, ex *Vitis vinifera*, La Central-Aplao (Castilla), 21/08/2021, A Llacctas.

###### Remarks.

Marin-Loayza and Cisneros (1995) reported this species on *Annona cherimola* Mill., *Annona muricata* L., and *Mammea americana* L. in Peru. This is the first record of *C. rusci* found on avocados and grapes in Arequipa. It has also been found on avocados and grapes in Israel (Ben-Dov 1970b).

##### ﻿ Ceroplastes sinensis

Taxon classificationAnimaliaHemipteraCoccidae

Del Guercio, 1900

B727B9CC-533F-5F28-B1C7-B59AD859C7EE

Suppl. material 1: fig. S2E, F

###### Distribution.

Peru: Camana.

###### Examined material.

• 1 adult female, ex *Vitis vinifera*, Camana (Camana), 28/02/2020, A Llacctas.

###### Remarks.

This is the first record of this species in Peru. It was found on grapes in Arequipa and has also been found on avocados in the Canary Islands of Spain (Carnero and Perez 1986) and Australia (Qin and Gullan 1994).

##### ﻿ Coccus hesperidum

Taxon classificationAnimaliaHemipteraCoccidae

Linnaeus, 1758

1233AC7A-7F15-5E5F-9A60-3D5ED9669D42

Suppl. material 1: fig. S3A, B

###### Distribution.

Peru: Arequipa, Camana, Caraveli, Castilla, Caylloma, Condesuyos, La Union.

###### Examined material.

• 1 adult female, ex *Persea americana*, Samuel Pastor (Camana), 29/06/2019, J Jihuallanca; • 14 adult females, ex *Persea americana*, San Juan de Chorunga-Rio Grande (Condesuyos), 30/11/2019, J Jihuallanca; • 4 adult females, ex *Persea americana*, Chaparra (Caraveli), 11/02/2020, J Jihuallanca; • 12 adult females, ex *Persea americana*, Andamayo-Aplao (Castilla), 14/03/2020, J Jihuallanca; • 3 adult females, ex *Persea americana*, El Pedregal-Majes (Caylloma), 26/08/2020, J Jihuallanca; • 7 adult females, ex *Persea americana*, La Joya (Arequipa), 28/08/2020, J Jihuallanca; • 2 adult females, ex *Persea americana*, Cotahuasi (La Union), 24/07/2022, J Jihuallanca; • 1 adult female, ex *Vitis vinifera*, Chaparra (Caraveli), 15/06/2019, A Llacctas; • 1 adult female, ex *Vitis vinifera*, Santa Isabel de Siguas (Arequipa), 23/07/2019, A Llacctas; • 1 adult female, ex *Vitis vinifera*, La Barrera-Yanaquihua (Condesuyos), 13/03/2020, A Llacctas; • 1 adult female, ex *Vitis vinifera*, Camana (Camana), 19/08/2021, A Llacctas; • 1 adult female, ex *Vitis vinifera*, Corire-Aplao (Castilla), 20/08/2021, A Llacctas; • 1 adult female, ex *Vitis vinifera*, Andamayo-Aplao (Castilla), 21/08/2021, A Llacctas; • 1 adult female, ex *Vitis vinifera*, La Central-Aplao (Castilla), 21/08/2021, A Llacctas.

###### Remarks.

Bederski (1969), and Beingolea (1967) reported this species on citrus in Peru. This is the first record of this species found on avocados and grapes in Arequipa.

##### ﻿ Coccus longulus

Taxon classificationAnimaliaHemipteraCoccidae

(Douglas, 1887)

5247B202-5433-568B-8210-497212DE6325

Suppl. material 1: fig. S3 C, D

###### Distribution.

Peru: Camana, Caraveli, Castilla, Condesuyos.

###### Examined material.

• 1 adult female, ex *Vitis vinifera*, Chaparra (Caraveli) 15/06/2019, • 1 adult female, ex *Vitis vinifera*, Samuel Pastor (Camana) 30/06/2019, A Llacctas; A Llacctas; • 2 adult females, ex *Vitis vinifera*, La Barrera-Yanaquihua (Condesuyos), 13/03/2020, A Llacctas; • 1 adult female, ex *Vitis vinifera*, Huancarqui (Castilla), 15/03/2020, A Llacctas.

###### Remarks.

This is the first record of this species in Peru. It was found on grapes in Arequipa, and has been found on grapes in India (Ali 1971), and New Zealand (Hodgson and Henderson 2000).

##### ﻿ Kilifia acuminata

Taxon classificationAnimaliaHemipteraCoccidae

(Signoret, 1873)

3614878E-0F64-589A-A722-0ABEEEBADDC8

Suppl. material 1: fig. S4A, B

###### Distribution.

Peru: Caraveli, Castilla, Condesuyos, Islay.

###### Examined material.

• 3 adult females, ex *Olea europaea*, San Juan de Chorunga-Rio Grande (Condesuyos), 28/06/2019, M Beltran; • 3 adult females, ex *Olea europaea*, Bella Union (Caraveli), 10/02/2020, M Beltran; • 3 adult females, ex *Olea europaea*, Acari (Caraveli), 10/02/2020, M Beltran; • 1 adult female, ex *Olea europaea*, Punta de Bombon (Islay), 29/02/2020, M Beltran; • 1 adult female, ex *Olea europaea*, La Central-Aplao (Castilla), 21/08/2021, M Beltran.

###### Remarks.

This is the first record of this species in Peru. It was found on olives in Arequipa. Nakahara (1981) reported it on avocados, and on other hosts of the Oleaceae family in Hawaii.

##### ﻿ Parasaissetia nigra

Taxon classificationAnimaliaHemipteraCoccidae

(Nietner, 1861)

3F45D0C0-FBFA-5F50-87CA-A198204D2FEE

Suppl. material 1: fig. S4C, D

###### Distribution.

Peru: Arequipa, Camana, Caraveli, Castilla, Condesuyos.

###### Examined material.

• 1 adult female, ex *Persea americana*, Camana (Camana), 29/06/2019, J Jihuallanca; • 1 adult female, ex *Persea americana*, Santa Isabel de Siguas (Arequipa), 23/07/2020, J Jihuallanca; • 3 adult females, ex *Persea americana*, Huancarqui (Castilla), 21/08/2021, J Jihuallanca; • 1 adult female, ex *Vitis vinifera*, Camana (Camana), 28/02/20, A Llacctas; • 2 adult females, ex *Vitis vinifera*, La Barrera-Yanaquihua (Condesuyos), 13/03/20, A Llacctas; • 1 adult female, ex *Vitis vinifera*, Vitor (Arequipa), 27/08/20, A Llacctas; • 1 adult female, ex *Vitis vinifera*, Chaparra (Caraveli), 17/08/21, A Llacctas; • 1 adult female, ex *Vitis vinifera*, Huancarqui (Castilla), 21/08/21, A Llacctas.

###### Remarks.

This is the first record of this species found on avocados and grapes in Arequipa. Marin-Loayza and Cisneros (1979) reported it on avocados and grapes from Peru. Elsebaay and Ahmed (2017) reported it on olives in Egypt, and Ben-Dov (1978) list grapes and avocados among its hosts.

##### ﻿ Protopulvinaria pyriformis

Taxon classificationAnimaliaHemipteraCoccidae

(Cockerell, 1894)

14A144D3-0959-5E37-BC49-024135A4E183

Suppl. material 1: fig. S4E, F

###### Distribution.

Peru: Camana, Castilla.

###### Examined material.

• 5 adult females, ex *Persea americana*, Samuel Pastor (Camana), 28/02/2020, J Jihuallanca; • 2 adult females, ex *Persea americana*, Huancarqui (Castilla), 15/03/2020, J Jihuallanca.

###### Remarks.

Couturier et al. (1997) reported this species on fruit trees of the Myrtaceae family in Pucallpa, Peru. This is the first record of this species found on avocados in Arequipa.

##### ﻿ Pulvinaria psidii

Taxon classificationAnimaliaHemipteraCoccidae

Maskell, 1893

FFFEEDB0-3319-53A9-BD04-0C0EB3A6787F

Suppl. material 1: fig. S4G, H

###### Distribution.

Peru: Arequipa.

###### Examined material.

• 5 adult females, ex *Olea europaea*, Vitor (Arequipa), 24/07/2019, M Beltran; • 5 adult females, ex *Olea europaea*, Vitor (Arequipa), 27/08/2020, M Beltran.

###### Remarks.

This is the first record of this species in Peru; it was found on olives in Arequipa.

##### ﻿ Saissetia coffeae

Taxon classificationAnimaliaHemipteraCoccidae

(Walker, 1852)

4D79DAC7-15D4-57AA-8F78-7BE4C26AD734

Suppl. material 1: fig. S5A, B

###### Distribution.

Peru: Camana, Caraveli, Castilla, Caylloma, Condesuyos, Islay.

###### Examined material.

• 4 adult females, ex *Olea europaea*, Chaparra (Caraveli), 11/02/2020, M Beltran; • 1 adult female, ex *Olea europaea*, San Juan de Chorunga-Rio Grande (Condesuyos), 28/06/2019, M Beltran; • 5 adult females, ex *Olea europaea*, Samuel Pastor (Camana), 28/02/2020, M Beltran; • 4 adult females, ex *Olea europaea*, Quilca (Camana), 28/02/2020, M Beltran; • 5 adult females, ex *Olea europaea*, Punta de Bombon (Islay), 29/02/2020, M Beltran; • 2 adult females, ex *Olea europaea*, Ongoro-Aplao (Castilla), 14/03/2020, M Beltran; • 5 adult females, ex *Olea europaea*, Pampas de la Amistad- Majes (Caylloma), 28/08/2020, M Beltran.

###### Remarks.

Aguilar et al. (1980) reported *S. coffeae* on olives in Tacna, and Beingolea and Salazar (1970) on olives in Yauca, and Bella Union, as a “rare” species, that only causes serious problems in Yauca (Caraveli). Also, it has been reported as a minor pest of citrus trees (Beingolea 1967). Corseuil and Barbosa (1971) recorded this species on olives in Brazil, Hodgson and Henderson (2000) reported it on olives in New Zealand, and Nakahara (1981) reported it on grapes in the Hawaiian Islands.

##### ﻿ Saissetia neglecta

Taxon classificationAnimaliaHemipteraCoccidae

De Lotto, 1969

4616BC19-3BDF-5B19-B854-9E880EA5791C

Suppl. material 1: fig. S5C, D

###### Distribution.

Peru: Caraveli.

###### Examined material.

• 9 adult females, ex *Persea americana*, Chaparra (Caraveli), 15/06/2019, J Jihuallanca.

###### Remarks.

This is the first record of this species found in Peru; based on our collection of this on avocados in Arequipa. Kondo and Muñoz (2016) reported it on avocados in Colombia.

##### ﻿ Saissetia oleae

Taxon classificationAnimaliaHemipteraCoccidae

(Olivier, 1791)

820D9D0C-6CAA-5333-AE7B-2C606C3D4C16

Suppl. material 1: fig. S5E, F

###### Distribution.

Peru: Camana, Caraveli, Castilla, Caylloma, Islay.

###### Examined material.

• 2 adult females, ex *Olea europaea*, Camana (Camana), 30/06/2019, M Beltran; • 5 adult females, ex *Olea europaea*, Acari (Caraveli), 10/02/2020, M Beltran; • 5 adult adult females, ex *Olea europaea*, Chaparra (Caraveli), 10/02/2020, M Beltran; • 5 adult females, ex *Olea europaea*, Yauca (Caraveli), 11/02/2020, M Beltran; • 2 adult females, ex *Olea europaea*, Punta de Bombon (Islay), 29/02/2020, M Beltran; • 2 adult females, ex *Olea europaea*, Ongoro-Aplao (Castilla), 14/03/2020, M Beltran; • 4 adult females, ex *Olea europaea*, Pampas de la amistad-Majes (Caylloma), 28/08/2020, M Beltran.

###### Remarks.

Gonzalez (1966) and Aguilar et al. (1980) reported this species on olives in Peru, Beingolea (1967) recorded as an occasional pest casual in citrus orchards in Peru, and Beingolea and Salazar (1970) observed this species on olives from several localities in Arequipa.

#### ﻿Diaspididae

##### ﻿ Aspidiotus nerii

Taxon classificationAnimaliaHemipteraCoccidae

Bouche, 1833

95D86235-6C42-5764-8CF7-A24CC007EE72

Suppl. material 1: fig. S6A, B

###### Distribution.

Peru: Arequipa, Camana, Caraveli, Castilla, Caylloma, Condesuyos, Islay, La Union.

###### Examined material.

• 2 adult females, ex *Persea americana*, Corire (Castilla), 12/07/2019, J Jihuallanca; • 1 adult female, ex *Persea americana*, Caramba-Chaparra (Caraveli), 11/02/2020, J Jihuallanca; • 7 adult females, ex *Persea americana*, Chichas (Condesuyos), 12/03/2020, J Jihuallanca; • 22 adult females, ex *Persea americana*, Uchumayo (Arequipa), 28/08/2020, J Jihuallanca; • 2 adult females, ex *Persea americana*, Cotahuasi (La Union), 24/07/2022, J Jihuallanca; • 2 adult females, ex *Vitis vinifera*, Caraveli (Caraveli), 12/02/2020, A Llacctas; • 4 adult females, ex *Vitis vinifera*, Quilca (Camana), 28/02/2020, A Llacctas; • 1 adult female, ex *Vitis vinifera*, Uchumayo (Arequipa), 04/03/2020, • 1 adult female, ex *Vitis vinifera*, Chichas (Condesuyos), 12/03/2020; A Llacctas; • 1 adult female, ex *Vitis vinifera*, Chaupo-Quechualla (La Union), 24/07/22, A Llacctas; • 2 adult females, ex *Olea europaea*, Vitor (Arequipa), 24/07/2019, M Beltran; • 2 adult females, ex *Olea europaea*, Bella Union (Caraveli) 10/02/2020, M Beltran; • 5 adult females, ex *Olea europaea*, Yauca (Caraveli) 11/02/2020, M Beltran; • 4 adult females, ex *Olea europaea*, Quilca (Camana) 28/02/2020, M Beltran; • 1 adult female, ex *Olea europaea*, Cocachacra (Islay), 29/02/2020, M Beltran; • 3 adult females, ex *Olea europaea*, Chichas (Condesuyos) 12/03/2020, M Beltran; • 1 adult female, ex *Olea europaea*, Ongoro-Aplao (Castilla), 14/03/2020, M Beltran; • 2 adult females, ex *Olea europaea*, Pampas de la amistad-Majes (Caylloma), 28/08/2020, M Beltran.

###### Remarks.

Bartra (1976) reported this species as *A. hederae* (Comstock, 1883), a junior synonymy of *Aspidiotus nerii* Bouche, 1883 on avocados in Peru, and Beingolea and Salazar (1970) reported it on olives in Peru.

##### ﻿ Chrysomphalus dictyospermi

Taxon classificationAnimaliaHemipteraCoccidae

(Morgan, 1889)

EBE67443-1AA7-5CE6-B38C-286EA1A60382

Suppl. material 1: fig. S6C, D

###### Distribution.

Peru: Arequipa, Camana, Caraveli, Castilla, Condesuyos.

###### Examined material.

• 7 adult females, ex *Persea americana*, Santa Isabel de Siguas (Arequipa), 23/07/2019, J Jihuallanca; • 18 adult females, ex *Persea americana*, San Juan de Chorunga-Rio Grande (Condesuyos), 29/11/2019, J Jihuallanca; • 13 adult females, ex *Persea americana*, Chaparra (Caraveli), 11/02/2020, J Jihuallanca; • 7 adult females, ex *Persea americana*, Samuel Pastor (Camana), 28/02/2020, J Jihuallanca; • 7 adult females, ex *Persea americana*, Andamayo-Aplao (Castilla), 14/03/2020, J Jihuallanca; • 1 adult female, ex *Vitis vinifera*, Samuel Pastor (Camana), 30/06/2019, A Llacctas; • 1 adult female, ex *Vitis vinifera*, La Barrera-Yanaquihua (Condesuyos), 13/03/20, A Llacctas; • 1 adult female, ex *Vitis vinifera*, Uchumayo (Arequipa), 04/03/20, A Llacctas; • 1 adult female, ex *Vitis vinifera*, Luchea-Aplao (Castilla), 14/03/20, A Llacctas; • 2 adult females, ex *Vitis vinifera*, La Central-Aplao (Castilla) 14/03/20, A Llacctas.

###### Remarks.

Beingolea (1967) and Beingolea (1969a) reported *C. dictyospermi* on citrus in Peru. Nuñez (2008) reported that intense infestations have occurred in avocado trees in the southern coast of Peru. Our study found this species on avocados and grapes in Arequipa.

##### ﻿ Fiorinia fioriniae

Taxon classificationAnimaliaHemipteraCoccidae

(Targioni Tozzetti, 1867)

1E1D5323-A6F0-566C-842B-6957F5F8C8DB

Suppl. material 1: fig. S6E, F

###### Distribution.

Peru: Camana, Caraveli, Castilla, Islay.

###### Examined material.

• 7 adult females, ex *Persea americana*, Corire (Castilla), 12/07/2019, J Jihuallanca; • 4 adult females, ex *Persea americana*, Camana (Camana), 29/06/2019, J Jihuallanca; • 1 adult female, ex *Olea europaea*, Acari (Caraveli), 10/02/2020, M Beltran; • 4 adult females, ex *Olea europaea*, Cocachacra (Islay), 29/02/2020, M Beltran.

###### Remarks.

Collantes and Rodriguez (2022) reported it on avocados in Valle de Cañete, Lima, Peru. We found this species on avocados, and this is the first record on olives from Arequipa.

##### ﻿ Furchadaspis zamiae

Taxon classificationAnimaliaHemipteraCoccidae

(Morgan, 1890)

092336DB-AB41-50DC-B475-97EF023E09DE

Suppl. material 1: fig. S6G, H

###### Distribution.

Peru: Caraveli.

###### Examined material.

• 7 adult females, ex *Olea europaea*, Bella Union (Caraveli), 10/02/2020, M Beltran; • 4 adult females, ex *Olea europaea*, Acari (Caraveli), 10/02/2020, M Beltran.

###### Remarks.

This is the first record of this species in Peru; it was found on olives in Caraveli in the Arequipa region.

##### ﻿ Hemiberlesia cyanophylli

Taxon classificationAnimaliaHemipteraCoccidae

(Signoret, 1869)

DD0045F5-BD83-57E8-81D2-580A3F7670CE

Suppl. material 1: fig. S7A, B

###### Distribution.

Peru: Arequipa, Camana, Caraveli, Castilla, Caylloma, Condesuyos.

###### Examined material.

• 7 adult females, ex *Persea americana*, San Juan de Chorunga-Rio Grande (Condesuyos), 28/06/2019, J Jihuallanca; • 5 adult females, ex *Persea americana*, Corire (Castilla), 12/07/2019, J Jihuallanca; • 1 adult female, ex *Persea americana*, Santa Rita de Siguas (Arequipa), 23/07/2019, J Jihuallanca; • 8 adult females, ex *Persea americana*, Caramba-Chaparra (Caraveli), 11/02/2020, J Jihuallanca; • 8 adult females, ex *Persea americana*, Samuel Pastor (Camana), 28/02/2020, J Jihuallanca; • 3 adult females, ex *Persea americana*, El Pedregal-Majes (Caylloma), 26/08/2020, J Jihuallanca; • 1 adult female, ex *Vitis vinifera*, Chaparra (Caraveli), 15/06/19, A Llacctas; • 1 adult female, ex *Vitis vinifera*, Camana (Camana), 28/02/2020, A Llacctas; • 3 adult females, ex *Vitis vinifera*, Pedregal-Majes (Caylloma), 26/08/20, A Llacctas; • 1 adult female, ex *Vitis vinifera*, La Joya (Arequipa), 27/08/20, A Llacctas; • 1 adult female, ex *Olea europaea*, Camana (Camana), 30/06/2019, M Beltran; • 4 adult females, ex *Olea europaea*, Ongoro-Aplao (Castilla), 14/03/2020, M Beltran; • 1 adult female, ex *Olea europaea*, Yauca (Caraveli), 12/02/2020, M Beltran.

###### Remarks.

Beingolea and Salazar (1970) found this species on olives in two irrigation areas of Ica, Peru. Nuñez (2008) reported it on avocados, and olives in Peru, and Vasquez et al. (2002) reported it on guava (*Psidium guajava*) in Peru. Our study found it on avocados, olives, and grapes.

##### ﻿ Hemiberlesia lataniae

Taxon classificationAnimaliaHemipteraCoccidae

(Signoret, 1869)

15197ABB-3621-5F5E-8BB8-CE62A7ECF539

Suppl. material 1: fig. S7C, D

###### Distribution.

Peru: Arequipa, Camana, Caraveli, Castilla, Caylloma, Condesuyos, Islay, La Union.

###### Examined material.

• 9 adult females, ex *Persea americana*, El Pedregal-Majes (Caylloma), 22/07/2019, J Jihuallanca; • 12 females, ex *Persea americana*, Santa Isabel de Siguas (Arequipa), 23/07/2019, J Jihuallanca; • 3 adult females, ex *Persea americana*, San Juan de Chorunga-Rio Grande (Condesuyos), 29/11/2019, J Jihuallanca; • 10 adult females, ex *Persea americana*, Caraveli (Caraveli), 12/02/2020, J Jihuallanca; • 5 adult females, ex *Persea americana*, Andamayo-Aplao (Castilla), 14/03/2020, J Jihuallanca; • 1 adult female, ex *Persea americana*, Camana (Camana), 28/02/2020, J Jihuallanca; • 1 adult female, ex *Persea americana*, Cotahuasi (La Union), 24/07/2022, J Jihuallanca; • 1 adult female, ex *Vitis vinifera*, Ocoña (Camana), 27/02/2020, A Llacctas; • 4 adult females, ex *Vitis vinifera*, Camana (Camana), 28/02/20, A Llacctas; • 1 adult female, ex *Vitis vinifera*, La Barrera-Yanaquihua (Condesuyos), 14/03/20, A Llacctas; • 1 adult female, ex *Vitis vinifera*, Huatiapilla-Aplao (Castilla), 14/03/20, A Llacctas; • 1 adult female, ex *Vitis vinifera*, Sotillo-Vitor (Arequipa), 27/08/20, A Llacctas; • 2 adult females, ex *Vitis vinifera*, Caraveli (Caraveli), 18/08/21, A Llacctas; • 2 adult females, ex *Vitis vinifera*, Chaupo (La Union), 24/07/22, A Llacctas; • 2 adult females, ex *Olea europaea*, Bella Union (Caraveli), 10/02/2020, M Beltran; • 2 adult females, ex *Olea europaea*, Quilca (Camana), 28/02/2020, M Beltran; • 5 adult females, ex *Olea europaea*, Punta de Bombon (Islay), 29/02/2020, M Beltran; • 4 adult females, ex *Olea europaea*, Cocachacra (Islay), 29/02/2020, M Beltran; • 1 adult female, ex *Olea europaea*, Chichas (Condesuyos), 12/03/2020, M Beltran; • 3 adult females, ex *Olea europaea*, Ongoro-Aplao (Castilla), 14/03/2020, M Beltran.

###### Remarks.

Gonzalez (1966) reported *H. lataniae* on grapes from Arequipa. Beingolea and Salazar (1970) reported it in great quantities on olives from Arequipa. Nuñez (2008) reported on avocados from Peru. Our study found it on avocados from Arequipa.

##### ﻿ Hemiberlesia palmae

Taxon classificationAnimaliaHemipteraCoccidae

(Cockerell, 1893)

296BEBF7-E2FB-5A7D-9B80-70C70EEF5618

Suppl. material 1: fig. S7E, F

###### Distribution.

Peru: Camana, Caraveli, Castilla, Caylloma, Islay.

###### Examined material.

• 1 adult female, ex *Olea europaea*, Pampas De La Amistad-Majes (Caylloma), 28/08/2020, • 7 adult females, ex *Olea europaea*, Bella Union (Caraveli), 10/02/2020, M Beltran; • 4 adult females, ex *Olea europaea*, Acari (Caraveli), 10/02/2020, M Beltran; • 2 adult females, ex *Olea europaea*, Quilca (Camana), 28/02/2020, M Beltran; • 1 adult female, ex *Olea europaea*, Ongoro-Aplao (Castilla), 14/03/2020, M Beltran; • 1 adult female, ex *Olea europaea*, Punta de Bombon (Islay), 29/02/2020, M Beltran.

###### Remarks.

Vasquez et al. (2002) reported it on *Psidium guajava* in the Peruvian Amazonia. Our study found it on olives in Arequipa. Gonzalez and Charlin (1968), and Aguilera et al. (1981) reported it on avocados, and olives in Chile.

##### ﻿ Hemiberlesia rapax

Taxon classificationAnimaliaHemipteraCoccidae

(Comstock, 1881)

7333B6BC-6D7F-54F1-8E08-2DB7C28276E3

Suppl. material 1: fig. S7G, H

###### Distribution.

Peru: Arequipa, Camana, Caraveli, Castilla, Condesuyos.

###### Examined material.

• 1 adult female, ex *Vitis vinifera*, Caraveli (Caraveli), 12/02/2020; • 1 adult female, ex *Vitis vinifera*, Ocoña (Camana), 27/02/20, A Llacctas; • 4 adult females, ex *Vitis vinifera*, Quilca (Camana), 28/02/20, A Llacctas; • 4 adult females, ex *Vitis vinifera*, Uchumayo (Arequipa), 04/03/20, A Llacctas; • 1 adult female, ex *Vitis vinifera*, Corire-Uraca (Castilla), 15/03/20, A Llacctas; • 2 adult females, ex *Vitis vinifera*, La Barrera-Yanaquihua (Condesuyos), 13/03/20, A Llacctas; • 1 adult female, ex *Vitis vinifera*, Camana (Camana), 19/08/21, A Llacctas.

###### Remarks.

Nakahara (1982) reported this species from Peru. Beingolea and Salazar 1970 reported it on olives in Peru. Our study found it on grapes in the Arequipa region.

##### ﻿ Melanaspis squamea

Taxon classificationAnimaliaHemipteraCoccidae

Ferris, 1943

A2528E98-4971-5426-995B-64161C85F66A

Suppl. material 1: fig. S8A, B

###### Distribution.

Peru: Condesuyos.

###### Examined material.

1 adult female, ex *Persea americana*, San Juan de Chorunga-Rio Grande (Condesuyos), 28/06/2020, J Jihuallanca.

###### Remarks.

This is the first record of this species in Peru; it was found on avocados in Arequipa. Deitz and Davidson (1986) in their revision of the genus *Melanaspis* in North America listed *M. deklei*, *M. nigropunctata*, and *M. squamea* as associated with avocados and *M. odontoglossi* as the only species known from Peru. *Melanaspis squamea* is the only species in this group that lacks perivulvar pores and has transverse, dark sclerotized bars on the pygidium.

##### ﻿ Oceanaspidiotus spinosus

Taxon classificationAnimaliaHemipteraCoccidae

(Comstock, 1883)

80B950AD-08D2-5BC7-9AFC-B60DADAAC31A

Suppl. material 1: fig. S8C, D

###### Distribution.

Peru: Caraveli, Castilla, Condesuyos.

###### Examined material.

1 adult female, ex *Vitis vinifera*, Huancarqui (Castilla), 12/07/19, A Llacctas; 1 adult female, ex *Vitis vinifera*, La Barrera-Yanaquihua (Condesuyos), 13/03/20, A Llacctas; 1 adult female, ex *Vitis vinifera*, Chaparra (Caraveli), 17/08/2021, A Llacctas.

###### Remarks.

Nakahara (1982) reported *O. spinosus* in Peru. Our study found this species on grapes in Arequipa. Ferris (1938) reported on avocados and grapes in the United States, and Gerson and Zor (1973) reported it on avocados in Israel.

##### ﻿ Pinnaspis aspidistrae

Taxon classificationAnimaliaHemipteraCoccidae

(Signoret, 1869)

8796D235-690A-59CB-9978-123E05439209

Suppl. material 1: fig. S9A, B

###### Distribution.

Peru: Camana.

###### Examined material.

• 5 adult females, ex *Persea americana*, Camana 28/02/2020, J Jihuallanca; • 5 adult females, ex *Vitis vinifera*, Camana, 19/08/2021, A Llacctas.

###### Remarks.

Marin-Loayza and Cisneros (1982) reported this species on avocados from the central coast of Peru. Collantes and Rodriguez (2022) recorded *P. aspidistrae* on avocados in Valle de Cañete, Lima, Peru. Our study found this species on avocados, and grapes from Arequipa. Wolff et al. (2018) reported it on olives in Brazil.

##### ﻿ Pinnaspis strachani

Taxon classificationAnimaliaHemipteraCoccidae

(Cooley, 1899)

404C233E-53C4-509F-9288-2556B86811E8

Suppl. material 1: fig. S9C, D

###### Distribution.

Peru: Camana.

###### Examined material.

• 5 adult females, ex *Olea europaea*, Camana (Camana), 30/06/2019, M Beltran; • 6 adult females, ex *Olea europaea*, Quilca (Camana), 28/02/2020, M Beltran.

###### Remarks.

Marin-Loayza and Cisneros (1982) reported this species on olives in Peru. Our study found *P. strachani* on olives from Arequipa, and Wolff (2014) reported it on olives in Brazil.

##### ﻿ Pseudischnaspis bowreyi

Taxon classificationAnimaliaHemipteraCoccidae

(Cockerell, 1893)

F4DBBE40-B602-57FA-AD28-3A6EA7A85120

Suppl. material 1: fig. S10A, B

###### Distribution.

Peru: Camana.

###### Examined material.

• 1 adult female, ex *Vitis vinifera*, Camana, 28/02/2020, A Llacctas.

###### Remarks.

Miller et al. (1984) reported this species on several hosts in Peru, and Beingolea and Salazar (1970) reported it on olives in Peru. Our study found this species on grapes in Arequipa. Kondo and Muñoz (2016) reported on avocados in Colombia.

##### ﻿ Pseudoparlatoria parlatorioides

Taxon classificationAnimaliaHemipteraCoccidae

(Comstock, 1883)

9DDDC12B-1F8B-50AC-A9AF-1945CEDF3EA6

Suppl. material 1: fig. S10C, D

###### Distribution.

Peru: Arequipa, Camana, Caraveli, Castilla, Condesuyos, La Union.

###### Examined material.

• 6 adult females, ex *Persea americana*, Caraveli (Caraveli) 12/02/2020, J Jihuallanca; • 17 adult females, ex *Persea americana*, Chichas (Condesuyos), 12/03/2020, J Jihuallanca; • 1 adult female, ex *Persea americana*, Camana (Camana), 28/02/2020, J Jihuallanca; • 6 adult females, ex *Persea americana*, Andamayo-Aplao (Castilla), 14/03/2020, J Jihuallanca; • 6 adult females, ex *Persea americana*, La Joya (Arequipa), 28/08/2020, J Jihuallanca; • 2 adult females, ex *Persea americana*, Cotahuasi (La Union), 24/07/2022, J Jihuallanca.

###### Remarks.

This represents the first record of this species in Peru, and the first record of the species on avocados in Arequipa. Kondo and Muñoz (2016), and Wolff (2008) reported it on avocados in Colombia and Brazil, respectively.

##### ﻿ Selenaspidus articulatus

Taxon classificationAnimaliaHemipteraCoccidae

(Morgan, 1889)

6925F325-5285-5138-A0D9-12086BB843EB

Suppl. material 1: fig. S11A, B

###### Distribution.

Peru: Castilla.

###### Examined material.

• 5 adult females, ex *Persea americana*, Huatiapilla-Aplao (Castilla), 14/03/2020, J Jihuallanca.

###### Remarks.

Beingolea and Salazar (1970) reported this species on olives, Beingolea (1967) on citrus, and Beingolea (1969b) on olives, and citrus in Peru. Our study found this species on avocados in the Arequipa region.

#### ﻿Eriococcidae

##### ﻿ Ovaticoccus peruvianus

Taxon classificationAnimaliaHemipteraCoccidae

(Granara de Willink and Diaz 2007)

8D73E932-E041-5CE6-9EC9-E381A1B19B47

Suppl. material 1: fig. S12A, B

###### Distribution.

Peru: Arequipa, Castilla, Condesuyos.

###### Examined material.

• 1 adult female, ex *Vitis vinifera*, Huancarqui (Castilla) 12/07/2019, A Llacctas; • 2 adult females, ex *Vitis vinifera*, La Barrera (Condesuyos), 13/03/20, A Llacctas; • 2 adult females, ex *Vitis vinifera*, Corire (Castilla), 20/08/20, A Llacctas; • 1 adult female, ex *Vitis vinifera*, La Central (Castilla), 21/08/21, A Llacctas; • 2 adult females, ex *Vitis vinifera*, Vitor (Arequipa), 22/08/21, A Llacctas.

###### Remarks.

Granara de Willink and Diaz (2007) described this species as *Oregmopyga peruviana* on grapes in La Libertad, Lima, Ica, Arequipa, Moquegua, and Tacna, Peru, and stated that it greatly affects grapes in the Majes Valley, Arequipa. Miller and Stocks (2022) synonymized *Oregmopyga* with *Ovaticoccus*, which transferred this species to the genus *Ovaticoccus*, and reported it on *Beaucarnea* sp. from Mexico, and grass from Oklahoma (USA).

#### ﻿Ortheziidae

##### ﻿ Praelongorthezia olivicola

Taxon classificationAnimaliaHemipteraCoccidae

(Beingolea, 1965)

B1D843A2-2521-5E47-9DA5-0F6469995A0D

Suppl. material 1: fig. S13A, B

###### Distribution.

Peru: Camana, Caraveli, Islay.

###### Examined material.

• 5 adult females, ex *Olea europaea*, Ocoña (Camana), 29/06/2019, M Beltran; 5 adult females, ex *Olea europaea*, Punta de Bombon (Islay), 30/06/2019, M Beltran; • 5 adult females, ex *Olea europaea*, Bella Union (Caraveli), 10/02/2020, M Beltran; 5 adult females, ex *Olea europaea*, Yauca (Caraveli), 11/02/2020, M Beltran.

###### Remarks.

Beingolea (1965), and Beingolea and Salazar (1970), reported this species as *Orthezia olivicola*Beingolea 1965, a junior synonym of *Praelongorthezia olivicola* (Beingolea, 1965) on olives from Yauca, Acari, Bella Union, Chaparra, Caraveli, and Camana, Arequipa. Our study found it in several of these same sites.

#### ﻿Pseudococcidae

##### ﻿ Planococcus citri

Taxon classificationAnimaliaHemipteraCoccidae

(Risso, 1813)

47C7C0F5-9F26-5FAA-8595-E85AF0335FE1

Suppl. material 1: fig. S14A, B

###### Distribution.

Peru: Condesuyos.

###### Examined material.

• 1 adult female, ex *Persea americana*, San Juan de Chorunga-Rio Grande (Condesuyos), 29/11/2019, J Jihuallanca.

###### Remarks.

This species was previously reported on *Vitis vinifera* (Salazar 1972), in citrus orchards (Bederski 1969), on avocados (Ben-Dov 1994), and on apple, *Malus domestica* Borkh. in Mala valley (De la Cruz 1996) in Peru. Our study found it on avocados in Arequipa.

##### ﻿ Planococcus ficus

Taxon classificationAnimaliaHemipteraCoccidae

(Signoret, 1875)

8ECB6421-2A13-5093-A217-7A173A884875

Suppl. material 1: fig. S14C, D

###### Distribution.

Peru: Arequipa, Castilla, Caylloma.

###### Examined material.

• 1 adult female, ex *Vitis vinifera*, 24/07/2019, La Joya (Arequipa), A Llacctas; • 1 adult female, ex *Vitis vinifera*, 15/03/20, Corire-Uraca (Castilla), A Llacctas; • 1 adult female, ex *Vitis vinifera*, 27/08/20, La Joya (Arequipa), A Llacctas; • 2 adult females, ex *Vitis vinifera*, 22/01/21, El Pedregal-Majes (Caylloma), A Llacctas; • 1 adult female, ex *Vitis vinifera*, 22/08/2021, La Joya (Arequipa), A Llacctas.

###### Remarks.

This is the first report of this species in Peru; it was found on grapes in Arequipa, and was more prevalent in samples from Caylloma (Arequipa). It has been reported on a variety of hosts, especially on grapevines in the Palaearctic, Afrotropical, and Neotropical regions (Cox 1989).

##### ﻿ Pseudococcus longispinus

Taxon classificationAnimaliaHemipteraCoccidae

(Targioni Tozzetti, 1867)

0AF13C6E-CE2E-562F-9A2E-080FF1E89F54

Suppl. material 1: fig. S14E, F

###### Distribution.

Peru: Camana, Caraveli, Castilla, Condesuyos, Islay.

###### Examined material.

• 6 adult females, ex *Persea americana*, Camana (Camana), 29/06/2019, J Jihuallanca; • 1 adult female, ex *Persea americana*, Huancarqui-Aplao (Castilla), 15/03/2020, J Jihuallanca; • 1 adult female, ex *Vitis vinifera*, La Barrera-Yanaquihua (Condesuyos), 13/03/20, A Llacctas; • 1 adult female, ex *Vitis vinifera*, Luchea- Aplao (Castilla), 14/03/20, A Llacctas; • 1 adult female, ex *Vitis vinifera*, Camana (Camana), 19/08/21, A Llacctas; • 5 adult females, ex *Olea europaea*, Bella Union (Caraveli), 10/02/2020, M Beltran; • 5 adult females, ex *Olea europaea*, Yauca (Caraveli), 12/02/2020, M Beltran; • 5 adult females, ex *Olea europaea*, Samuel Pastor (Camana), 28/02/2020, M Beltran; • 2 adult females, ex *Olea europaea*, Punta de Bombon (Islay), 29/02/2020, M Beltran; • 1 adult female, ex *Olea europaea*, La Central-Aplao (Castilla), 21/08/2021, M Beltran.

###### Remarks.

Salazar (1972) reported this species on olives in Peru, and Carnero and Perez (1986) recorded it on avocados in the Canary Islands, and Cox (1987) reported it on grapes in New Zealand. Our study found *P. longispinus* on avocados, grapes, and olives from Arequipa.

##### ﻿ Pseudococcus viburni

Taxon classificationAnimaliaHemipteraCoccidae

(Signoret, 1875)

4049E980-5D0A-59D4-A9F6-0D3A98457035

Suppl. material 1: fig. S14G, H

###### Distribution.

Peru: Caylloma.

###### Examined material.

• 5 adult females, *ex Vitis vinifera*, 22/04/2018, El Pedregal-Majes (Caylloma), A Llacctas.

###### Remarks.

Salazar (1972) reported this species as *Pseudococcus obscurus* Essig, 1909, a junior synonym of *Pseudococcus viburni* on *Ficus carica* in Peru. Ben-Dov (1994) reported it on grapes in Peru. Our study found it on grapes in Arequipa, from samples collected exclusively from Pedregal (Caylloma).

### ﻿Key to adult females of scale insects found on avocados, olives, and grapes in Arequipa, Peru

**Table:** 

1	In nature: species with a plate-like wax cover that can be lifted off exposing the insect underneath it; slide specimen: The posteriormost four abdominal segments (V–VIII) are fused together, forming the pygidium; legs absent; armored scales	**Diaspididae 5**
1b	In nature: species flat to globose, and without a plate-like wax cover that can be lifted off; slide specimen: abdominal segments not fused together forming a pygidium; legs present in all of the species included herein	**2**
2(1b)	In nature: most species included herein are broadly oval to round adult female often with a translucent wax cover, rarely covered with whitish wax, but sometimes with white cottony-like ovisac extending from the posterior of the body (in *Pulvinaria* and *Protopulvinaria*); slide specimens: anal opening not surrounded by rows of cells, but covered by a pair of opposing triangular shaped, sclerotized plates; soft scales	**Coccidae 19**
2b	In nature: species usually covered with whitish wax; most species elliptical to round; slide specimens: anal opening surrounded by rows of cells, and not covered by sclerotized plate (s)	**3**
3(2b)	In nature: adult female covered with rows of grooved, bleach white wax which extend past the posterior margin by approx. the length of the body; legs exceptionally large, flat, and angular similar to those of a crab in appearance; slide specimen: abdominal spiracles present (in addition to the 2 pairs of spiracles on the cephalothorax); antennae 8 segmented, terminating in a large spine; ostioles, circuli, and cerarii absent; one species included herein; ensign scales	**Ortheziidae**: ***Praelongorthezia olivicola* (Beingolea)**
3b	In nature: adult female usually covered by fine mealy or cottony, felt-like wax, which rarely extends past the posterior margin by the length of the body; legs not exceptionally large, flat and angular similar to a crab in appearance; slide specimen: abdominal spiracles absent (2 pairs of spiracles on the cephalothorax present); ostioles, circuli, and cerarii present in the pseudococcid species included herein, absent in eriococcid species; antennae 7–8 segmented, without a spine at its apex, in the species included herein	**4**
4(3b)	In nature: adult female long and slender, body completely covered in snow white, felt-like wax; slide specimen: ostioles, circuli, and cerarii absent; dorsum with many enlarged, often candy kiss or stove pipe shaped setae; antennae 7 segmented; one species included herein; felt scales	**Eriococcidae: *Ovaticoccus peruvianus* (Granara de Willink & Diaz)**
4b	In nature: adult female usually not as long and slender, body completely covered with mealy-like, white wax; dorsal setae flagellate and ostioles, circulus, and cerarii present in species included herein; mealybugs	**Pseudococcidae 29**
** Diaspididae **		
5(1)	Pygidium with 2-barred macroducts; second lobe (L2) bilobate (divided); anterior spiracles usually associated with disc pores; gland spines often present between pygidial lobes (Diaspidinae), body long and slender, ~2.0–2.5× as long as wide; except in *Furchadaspis zamiae* and *Pseudoparlatoria parlatorioides* which are 1.3× as long as wide	**Diaspidinae 6**
5b	Pygidium with 1-barred macroducts; L2 unilobate (not divided); anterior spiracles without disc pores; fringed plates present between pygidial lobes; body oval, round, or turbinate, ~1.2–1.3× as long as wide; except in *Pseudischnaspis bowreyi* which is bullet-shaped and 2.4× as long as wide	**Aspidiotinae 10**
6(5)	Body oval-shaped, 1.3× as long as wide; perivulvar pores present or absent	**7**
6b	Body long and slender, ~2.0–2.5× as long as wide; perivulvar pores present	**8**
7(6)	Perivulvar pores absent; much of body covered with rows of short ducts; anterior spiracles associated with disc pores; median lobes (L1) widely divergent; gland spines present between L1 lobes, not joined basally	***Furchadaspis zamiae* (Morgan)**
7b	Perivulvar pores present; body with sparsely scattered elongate ducts; anterior spiracles not associated with disc pores; median lobes (L1) not divergent; L1 lobes with “fish-tail-like” gland spines, joined basally	***Pseudoparlatoria parlatorioides* (Comstock)**
8(6b)	In nature: adult female pupillarial-form, encased in its last nymphal exuviae; slide specimen: L1 lobes widely separated, diverging; pygidial macroducts confined to marginal and submarginal areas; antenna horn-like with a long slender seta at its base in this species	***Fiorinia fioriniae* (Targioni-Tozzetti)**
8b	In nature: adult female not pupillarial, adult not encased in its last nymphal exuviae); slide specimen: L1 lobes closely appressed and not diverging; pygidial macroducts not confined to marginal and submarginal areas; antenna not horn-like	***Pinnaspis* 9**
9(8b)	In nature: scale cover white; slide specimen: dorsal pre-anal sclerosis (arched, dark sclerotization anterolateral to the anal opening) present	***Pinnaspis strachani* (Cooley)**
9b	In nature: scale cover brownish; slide specimen: dorsal pre-anal sclerosis absent	***Pinnaspis aspidistrae* (Signoret)**
10(5b)	Body bullet-shaped, parallel-sided, elongate, 2.4× as long as wide; paraphyses long and slender with a paraphysis arising between the L2 and L3 lobes	***Pseudischnaspis bowreyi* (Cockerell)**
10b	Body oval, round or turbinate, not parallel sided, 1.2–1.3× as long as wide; paraphyses not long and slender or absent	**11**
11(10b)	Prosoma with a marked constriction between mesothorax and metathorax; with a sharp spur at posterolateral apex of mesothorax; small paraphyses and 1 group of perivulvar pores on each side of pygidium; L3 widely separated from L2 (3× the width of L2)	***Selenaspidus articulatus* (Morgan)**
11b	Prosoma not with a marked constriction between mesothorax and metathorax and without a sharp spur; paraphyses and perivulvar pores variable; L3 not widely separated from L2 lobe	**12**
12(11b)	Pygidial paraphyses absent	**13**
12b	Pygidial paraphyses present	**14**
13(12)	Pygidial macroducts ~4–5× as long as wide; L3 pointed, and with crenulate margins; dorsal seta associated with outer corners of L2 and L3 slender, not thickened basally	***Aspidiotus nerii* Bouche**
13b	Pygidial macroducts ~10–12× as long as wide; L3 spinelike, and with smooth margin; dorsal setae associated with outer corners of L2 and L3, thickened and swollen basally	***Oceanaspidiotus spinosus* (Comstock)**
14(12b)	All paraphyses shorter than L1; all arising from lobe bases, absent from spaces between lobes	***Hemiberlesia* 15**
14b	At least some paraphyses are longer than L1, not all arising from lobe bases, at least one arising from within interlobular space	**18**
15(14)	Perivulvar pores absent	***Hemiberlesia rapax* (Comstock)**
15b	Perivulvar pores present	**16**
16(15b)	L2 and L3 lobes represented by short, hyaline spines; L1, with lateral and medial margin not parallel, slightly convergent	***Hemiberlesia lataniae* (Signoret)**
16b	L2 and L3 lobes longer, sclerotized and trident; L1, with lateral and medial margin parallel, not convergent	**17**
17(16b)	Anal opening relatively large (wider and longer than L1) and separated from bases of anal lobes by ≤ 2× its longitudinal diameter; pygidial plates obviously longer than L1 and elaborately fringed; plates anterior to L3 usually fringed on outer margin as well as at tip; L2 long and slender, usually pointed; cosmopolitan	***Hemiberlesia palmae* (Cockerell)**
17b	Anal opening relatively small (narrower and shorter than L1) and separated from bases of anal lobes by > 2× its longitudinal diameter; pygidial plates ≤ slightly longer than L1 and only moderately fringed; plates anterior to L3 lacking lateral fringes; L2 long and narrow, apex usually rounded	***Hemiberlesia cyanophylli* (Signoret)**
18(14b)	In nature: scale cover yellow-brown, primarily on leaves and fruit; slide specimen: pygidium long tapering with apex somewhat truncate with convex margins; third lobe (L3) and fourth lobe (L4) of pygidium mitten-shaped with fleshy filaments longer than lobes between them; perivulvar pores present;	***Chrysomphalus dictyospermi* (Morgan)**
18b	In nature: scale cover dark brown to black and usually found on leaves or bark; slide specimen: pygidium shorter, nearly rounded posteriorly; L3 and L4 short and wide with 3–5 crenulate and 2 short plates between them; perivulvar pores absent	***Melanaspis squamea* Ferris**
** Coccidae **		
19(2)	In nature: body dome-shaped, round with thick, opaque waxy cover; slide specimen: derm around anal plates with large heavily sclerotized area; spiracular opening with numerous (> 3), bullet-shaped setae	***Ceroplastes* 20**
19b	In nature: body elliptical, oval or round, flat or dome-shaped, with a thin, translucent waxy cover; slide specimen: derm around anal plates without a large, heavily sclerotized area; each spiracular opening with 3 slender setae in species included herein	**22**
20(19)	Antennae 7-segmented (3 segments before long 4^th^ segment); tibiotarsal sclerosis present; submargin with a band of filamentous ducts	***C. sinensis* Del Guercio**
20b	Antennae 6-segmented (2 segments before long 3^rd^ segment); tibiotarsal scleroses and a band of submarginal filamentous ducts present or absent	**21**
21(20b)	Tibiotarsal scleroses present; submargin without a band of filamentous ducts	***C. rusci* (L.)**
21b	Tibiotarsal sclerosis absent; submargin with a band of filamentous ducts	***C. floridensis* Comstock**
22(19b)	In nature: mature female very globose, dome-shaped, and dark brown or black, never associated with a white cottony ovisac protruding from the posterior end of the body; slide specimen: derm with polygonal reticulation; a submarginal band of tubular ducts present	**23**
22b	In nature: mature female nearly flat, pale brown in color, some species associated with a white cottony ovisac protruding from the posterior end of the body; slide specimen: derm smooth, without polygonal reticulations in species included herein; a submarginal band of tubular ducts present or absent	**25**
23(22)	Anal plates without a pair of large discal setae; derm with a pale oval area inside each polygonal section; dorsal setae cylindrical or capitate	***Parasaissetia nigra* (Nietner)**
23b	Anal plates with a pair of large discal setae; derm usually without a pale oval area inside each polygonal section; dorsal setae spine-like	***Saissetia* 24**
24(23b)	Cephalothorax with < 30 marginal setae between the anterior spiracular clefts; row of ventral tubular ducts with a slender inner filament only	***S. neglecta* De Lotto**
24b	Cephalothorax with 40–60 marginal setae between the anterior spiracular clefts; row of ventral tubular ducts of 2 types – 1 with inner filament as wide or wider than the duct, the other with slender inner filament	***S. coffeae* (Walker)**
25(22b)	In nature: mature females often with a white cottony mass ovisac protruding from their posterior end; slide specimen: submargin with a row of tubular ducts	**26**
25b	In nature: mature females without a white cottony mass ovisac protruding from their posterior end; slide specimen: submargin without a row of tubular ducts	**27**
26(25b)	Anal plates elongate, the anterior margin of each plate 4–5× longer than the posterior margin and located subcentrally with a long anal cleft; body pear-shaped; anterior spiracles not surrounded by a C-shaped sclerotization; dorsal setae long and fimbriate	***Protopulvinaria pyriformis* (Cockerell)**
26b	Anal plates nearly quadrate, anterior margin of each plate approx. as long as the posterior margin and located in the posterior 1/5 of the body, with a relatively short anal cleft; oval-shaped; anterior spiracles surrounded by a C-shaped sclerotization; dorsal setae short and fimbriate	***Pulvinaria psidii* Maskell**
27(25b)	Anal plates elongate ~1.3× as long as width, located in posterior 2/5 of the body with a long anal cleft; body oblong, asymmetrical; marginal setae short, dendritic; dorsal setae capitate	***Kilifia acuminata* (Signoret)**
27b	Anal plates nearly quadrate, anterior margin of each plate approx. as long as the posterior margin and located in the posterior 0.2 of the body, with a relatively short anal cleft; symmetrical, elliptical, or oval-shaped; marginal setae long, flagellate often with fringed apices; dorsal setae sword-shaped or curved	***Coccus* 28**
28(27b)	Body broadly oval, ~1.4× as long as wide and not tapering posteriorly; dorsal setae sword-shaped; anal plates without a pair of subdiscal setae	***C. hesperidum* L.**
28b	Body elliptical, ~2× as long as wide and tapering posteriorly; dorsal setae curved; anal plates with a pair of subdiscal setae	***C. longulus* (Douglas)**
** Pseudococcidae **		
29(4b)	With 18 pairs of cerarii with 3 cerarii above the level of each eye, all cerarii without auxiliary setae (except those on anal lobe); oral rim tubular ducts absent	***Planococcus* 30**
29b	With 17 pairs of cerarii with 2 cerarii above the level of each eye, all cerarii with auxiliary setae; oral rim tubular ducts present	***Pseudococcus* 31**
30(29)	Hind femora almost always with translucent pores; area between the antennae with < 5 tubular ducts	***Pl. ficus* (Signoret)**
30b	Hind femora never with translucent pores; area between the antennae with > 5 tubular ducts	***Pl. citri* (Risso)**
31(29b)	In nature: posterior end often with exceptionally long wax filaments; slide specimen: each segment of the lateral margin of the abdomen with 3 oral rim tubular ducts; multilocular pores present only around the vulva	***Ps. longispinus* (Targ.-Tozz.)**
31b	In nature: posterior end without exceptionally long wax filaments; slide specimen: each segment of the lateral margin of the abdomen with no > 1 oral rim tubular duct; multilocular pores extend up to the third abdominal segment	***Ps. viburni* (Signoret)**

## ﻿Discussion

### ﻿Diversity

We collected a total of 13,991 scale insects from three different crops. Diaspididae and Coccidae were the dominant families, indicating their widespread distribution and potential economic importance. In contrast, Pseudococcidae, Ortheziidae, and Eriococcidae were far less abundant. While the dominant families were consistently present across all crops, host specificity was observed among the less common families: Eriococcidae occurred only on *V. vinifera*, and Ortheziidae was exclusive to *O. europaea*. These findings suggest a generalist tendency among the dominant families, and a more specialized host association among the rarer ones.

This study reveals a significantly higher diversity of scale insects in the three surveyed crops in the Arequipa region, with 33 species identified. This represents a substantial increase compared to earlier records, which documented only five species between 1965 and 2007 (Beingolea 1965; Gonzalez 1966; Beingolea and Salazar 1970; Aguilar et al. 1980; Granara de Willink and Diaz 2007). The marked rise in recorded diversity may be attributed to improved sampling methods, increased research efforts, and possibly changes in agricultural practices, and environmental conditions that have favored the establishment of more species.

The high diversity of scale insects observed in this study has important implications for Integrated Pest Management (IPM). This is significant because a more complex insect community increases the risk of secondary pest emergence. Ecologically, this higher diversity suggests that interactions within the agroecosystem may influence community structure. This finding highlights the importance of continued monitoring of scale insects in the Arequipa region.

Several scale insect species exhibited clear patterns of host association, with some showing strong crop specificity. Six species were found exclusively on avocados, eight on olives, and nine on grapes, indicating a preference for particular hosts. In contrast, a smaller group of species demonstrated broader host ranges. Notably, four species including *A. nerii*, *H. cyanophylli*, *H. lataniae*, and *P. longispinus* were present on all three crops, suggesting a generalist nature. *Fiorinia fioriniae* occurred on both avocados and olives, while five species were shared between avocados and grapes. These observations highlight the coexistence of both specialist, and generalist scale insect species within these cropping systems

### ﻿Economic concerns

#### ﻿Armored scales

*Aspidiotus nerii* was found associated with all three studied host plants. This species is widely distributed across seven provinces in the Arequipa region. Beingolea and Salazar (1970), and Bartra (1976) reported *A. nerii* (as *Aspidiotus hederae*) on olives and avocados in Peru. The historical records indicated severe damage to branches, leaves, and fruit, with estimated yield losses of up to 8 kg per plant (Beingolea and Salazar 1970). Currently, no quantitative damage data are available for avocado and grape crops.

*Hemiberlesia lataniae* was found associated with the three hosts plants that were studied. The species was recorded on olives in five provinces, on grapes in six provinces, and on avocados in seven provinces, with the highest population abundance observed on grapes. Beingolea and Salazar (1970) reported *H. lataniae* (as *Aspidiotus lataniae*) on olives in Peru stating that the population increases were linked to environmental conditions such as high temperatures, low humidity, strong winds, and dry dust. Under these conditions, population densities rose significantly, which could potentially impact both the yield, and quality of the fruit (Gonzalez 1966). However, there is currently no available data on the extent of potential damage to avocados and grapes.

*Hemiberlesia cyanophylli* was found associated with both olives and avocados. In six provinces, avocado orchards were infested with this species. A similar association was reported in Mexico, where *H. cyanophylli* was the most abundant scale insect found on avocado fruits (Lazaro-Castellanos et al. 2021); as Nuñez (2008) observed, the continuous presence of the insect on the underside of avocado leaves does not generally cause severe damage, except in cases where the biological control agents are eliminated due to the use of agrochemicals. On olives, *H. cyanophylli* was detected in five provinces, although in low numbers. Previous research by Beingolea and Salazar (1970) noted the presence of *H. cyanophylli* recorded as an incipient population. In the El Pedregal area, local farmers identified it as a key pest, reporting that infestations caused at least a 20% reduction in the market value of affected fruits (R Bedregal, pers. comm., 30 June 2023).

*Chrysomphalus dictyospermi* was found associated with two of the studied host plants. On grapes, the species was observed in low abundance, whereas on avocados it was the most abundant, and widely distributed. This species is considered a significant pest of citrus, avocados, and a wide range of tropical, and subtropical plants (Miller and Davidson 2005).

#### ﻿Mealybugs

*Planococcus ficus* was the most abundant mealybug in the sampled localities of the provinces of Arequipa, and Caylloma in this study. According to Cox (1989) it is highly probable that this species will continue to spread to other areas where grapevines are cultivated. This species is considered a key pest of vineyards worldwide, with damage reported from South Africa (Walton and Pringle 2004; Walton et al. 2009), Tunisia (Mansour et al. 2011), Brazil (Morandi Filho et al. 2015), and Argentina (Schulze-Sylvester et al. 2021).

*Pseudococcus viburni* was collected in a specific locality in the Caylloma province. In Peru, Salazar (1972) reported it on *Ficus carica* from Callao, providing no further information regarding the phytosanitary implications. It is a polyphagous species recorded on 236 plant genera across 89 families (Garcia-Morales et al. 2016). Evidence suggests its origin is in South America, and it is considered a major pest in Chile (Correa et al. 2015), and Brazil (Pacheco da Silva et al. 2017).

*Planococcus citri* was observed at low abundance on avocados in a single locality in Condesuyos. This species was previously reported infesting vineyards in the Mala Valley of Lima, causing a high percentage of fruit damage (Salazar 1972). This insect is actually a secondary pest. It only becomes noticeable when the natural balance is disturbed (Beingolea 1967).

#### ﻿Soft scales

*Saissetia oleae* was observed at low abundance on olives, and appeared to be widely distributed across five provinces in the Arequipa. In early studies, Gonzalez (1966) was the first to report this species on olives in Peru, and Beingolea and Salazar (1970) later observed it in several areas of Arequipa, including Mejia, Valle de Tambo, and Bella Union, and considered it one of the most important species affecting olives. Aguilar et al. (1980) also documented its presence on olives in Peru.

#### ﻿Felt scales

Granara de Willink and Diaz (2007) described this species as *Oregmopyga peruviana* Granara de Willink & Diaz, 2007, a junior synonym of *Ovaticoccus peruvianus* based on specimens found on grapes (*Vitis vinifera*) in Lima, Peru, and reported as a pest in vineyards due to its ability to damage woody stems. Further research is needed to conduct comprehensive taxonomic, and ecological studies of eriococcids in vineyards along the Peruvian coast. Such studies are essential to improve our understanding of their distribution, and impact, which is critical for effective pest management, and biodiversity conservation in vineyard ecosystems. Our study found *Ovaticoccus peruvianus* associated with grapevines.

#### ﻿Ensign scales

The ortheziid *Praelongorthezia olivicola* has been found exclusively in association with olive trees, with its known distribution now extending to three provinces. Early studies have reported heavy infestations of *Praelongorthezia olivicola* (as *Orthezia olivicola* Beingolea, 1965) on olives in Yauca, Atico, Chaparra, Acari, and Tambo in the Arequipa region (Beingolea 1965). Although the affected areas in the Ilo Valley (Moquegua, Peru) have shown limited geographic expansion, the species remains a concern in this region. Additional distribution records have been documented in northern Chile (Aguilera and Graña 1976). According to Beingolea (1965), the type of host plant attacked by this species largely depends on the accessibility of the host, suggesting that host selection may be opportunistic rather than highly specific. Beingolea (1965) also noted instances of taxonomic confusion in the literature, where *P. olivicola* was mistakenly identified as *Insignorthezia insignis* (Browne, 1887). However, slide-mounted specimens of the earlier studies were not available for examination.

## ﻿Conclusions

Armored scales were the most abundant group found in our study, accounting for 45% of all specimens, followed by soft scales at 36%. Mealybugs, and other less common families such as felt scales and ensign scales each contributed a smaller fraction (≤12% each), reflecting lower prevalence.

The Margalef index revealed that *V. vinifera* supports the richest scale insect community, indicating greater species richness compared to *P. americana* and *O. europaea*. The Shannon-Wiener and Simpson’s indices confirmed high overall diversity among the crops, with *V. vinifera* showing the most diverse, and evenly distributed scale insect community, while species dominance remained relatively uniform across all hosts. Evenness values suggest a fairly balanced distribution of individuals among species across all crops, with only minor variation, pointing to a moderately uniform community structure. The Berger-Parker index results further support the absence of dominant species, indicating a well-balanced scale insect composition within each agroecosystem.

The armored scales *A. nerii* and *H. lataniae* were the most widespread, occurring on all host plants. *Chrysomphalus dictyospermi*, although present in low numbers on *V. vinifera*, was the most dominant armored scale species on *P. americana*. Among the mealybugs, *P. ficus* stood out as the most significant pest on *V. vinifera*. In contrast, some species showed strong host specificity: the eriococcid *O. peruvianus* was found only on *V. vinifera*, while the ortheziid *P. olivicola* was restricted to *O. europaea*.

The findings of this study provide essential baseline data for monitoring scale insects in Arequipa region, and may be used in the development of effective control strategies.

## Supplementary Material

XML Treatment for Ceroplastes floridensis

XML Treatment for Ceroplastes rusci

XML Treatment for Ceroplastes sinensis

XML Treatment for Coccus hesperidum

XML Treatment for Coccus longulus

XML Treatment for Kilifia acuminata

XML Treatment for Parasaissetia nigra

XML Treatment for Protopulvinaria pyriformis

XML Treatment for Pulvinaria psidii

XML Treatment for Saissetia coffeae

XML Treatment for Saissetia neglecta

XML Treatment for Saissetia oleae

XML Treatment for Aspidiotus nerii

XML Treatment for Chrysomphalus dictyospermi

XML Treatment for Fiorinia fioriniae

XML Treatment for Furchadaspis zamiae

XML Treatment for Hemiberlesia cyanophylli

XML Treatment for Hemiberlesia lataniae

XML Treatment for Hemiberlesia palmae

XML Treatment for Hemiberlesia rapax

XML Treatment for Melanaspis squamea

XML Treatment for Oceanaspidiotus spinosus

XML Treatment for Pinnaspis aspidistrae

XML Treatment for Pinnaspis strachani

XML Treatment for Pseudischnaspis bowreyi

XML Treatment for Pseudoparlatoria parlatorioides

XML Treatment for Selenaspidus articulatus

XML Treatment for Ovaticoccus peruvianus

XML Treatment for Praelongorthezia olivicola

XML Treatment for Planococcus citri

XML Treatment for Planococcus ficus

XML Treatment for Pseudococcus longispinus

XML Treatment for Pseudococcus viburni

## ﻿Additional information

### Use of AI

No use of AI was reported.

### Author ORCIDs

Maholy Beltran-Moreno https://orcid.org/0000-0003-4945-4233

Anali Llacctas https://orcid.org/0009-0005-8207-6627

Javier Huanca https://orcid.org/0000-0002-6115-966X

Gregory Evans https://orcid.org/0000-0002-2165-3292

### Data availability

All of the data that support the findings of this study are available in the main text or Supplementary Information.

## Supplementary materials

Supplementary material 1 Additional information Data type pdf Explanation note **fig. S1** Map of the Arequipa region with the eight provinces. **fig. S2** Microphotography of *Ceroplastes floridensis*. **fig. S3** Microphotography of *Coccus hesperidum*. **fig. S4** Microphotography of *Kilifia acuminata*. **fig. S5** Microphotography of *Saissetia coffeae*. **fig. S6** Microphotography of *Aspidiotus nerii*. **fig. S7** Microphotography of *Hemiberlesia cyanophylli*. **fig. S8** Microphotography of *Melanaspis* sp. **fig. S9** Microphotography of *Pinnaspis aspidistrae*. **fig. S10** Microphotography of *Pseudischnaspis bowreyi*. **fig. S11** Microphotography of *Selenaspidus articulatus*. **fig. S12** Microphotography of *Ovaticoccus peruvianus*. **fig. S13** Microphotography of *Praelongorthezia olivicola*. **fig. S14** Microphotography of *Planococcus citri*. https://binary.pensoft.net/file/1453022This dataset is made available under the Open Database License (http://opendatacommons.org/licenses/odbl/1.0/). The Open Database License (ODbL) is a license agreement intended to allow users to freely share, modify, and use this Dataset while maintaining this same freedom for others, provided that the original source and author(s) are credited.Maholy Beltran, Anali Llacctas, Javier Huanca
