# Supplementary material for: ﻿A survey of scale insects (Hemiptera, Coccoidea) on avocados, olives, and grapes in the Peruvian region of Arequipa
Source: Zookeys. 2025 Oct 28;1257:91–125. doi: 10.3897/zookeys.1257.163722 (PMC12587173; doi:10.3897/zookeys.1257.163722)
Supplement: Supplementary material 1 — Additional information [file zookeys-1257-091_article-163722__-s001.pdf]

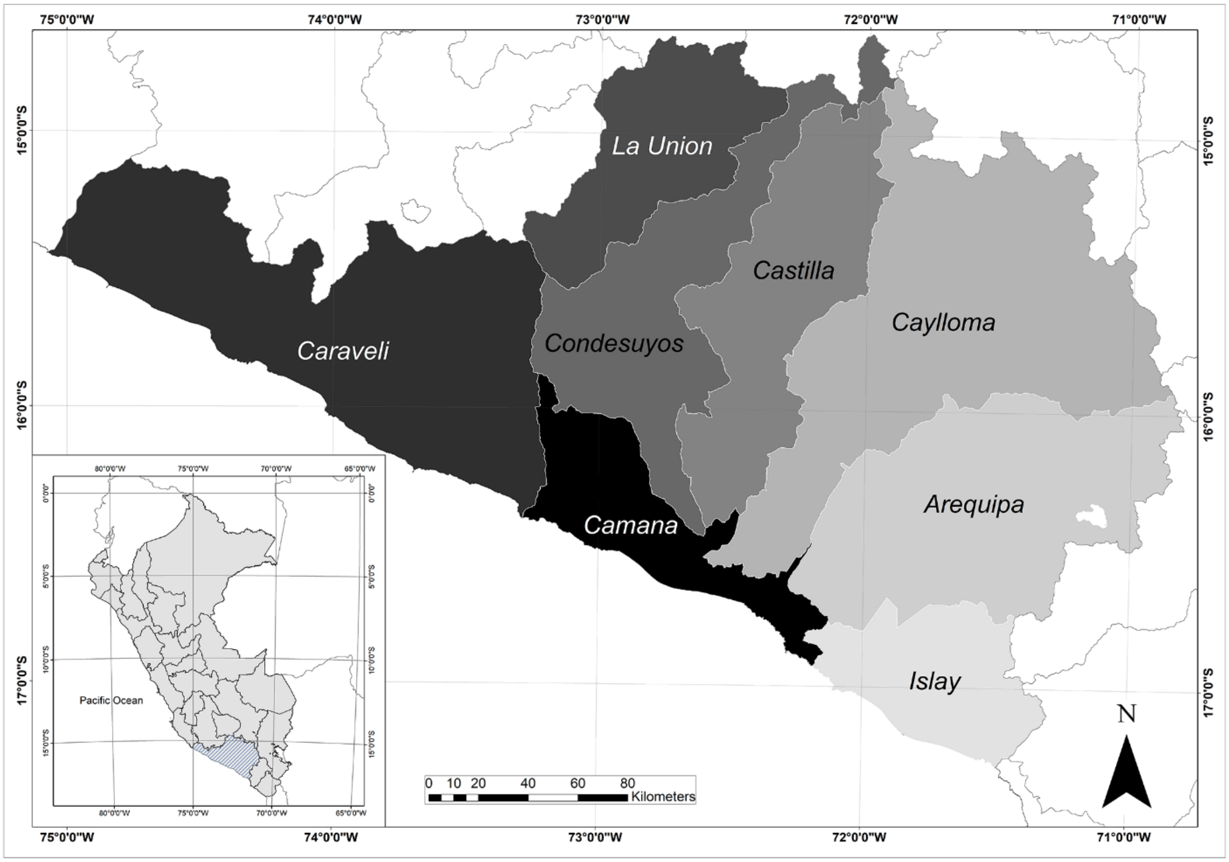

Figure S1. Map of the Arequipa region with the eight provinces.

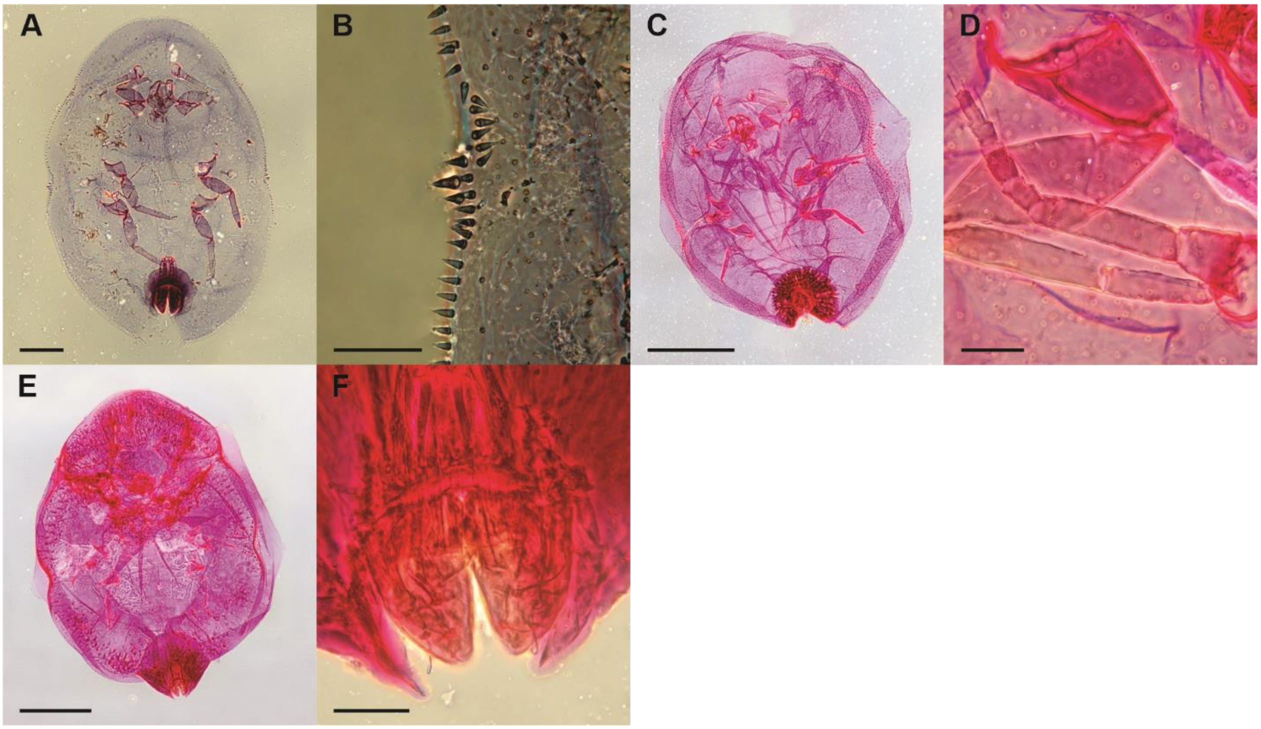

Figure S2. Microphotography of *Ceroplastes floridensis*, **A**: Body, **B:** Stigmatic setal arrangement on three rows; *Ceroplastes rusci*, **C**: Body, D: Stigmatic setal arrangement in two rows; *Ceroplastes sinensis*, **E**: Body, **F**. Two subapical seta on anal plates. Scale bars: 50 µm (B, D, F), 200 µm (A), 500 µm (C, E).

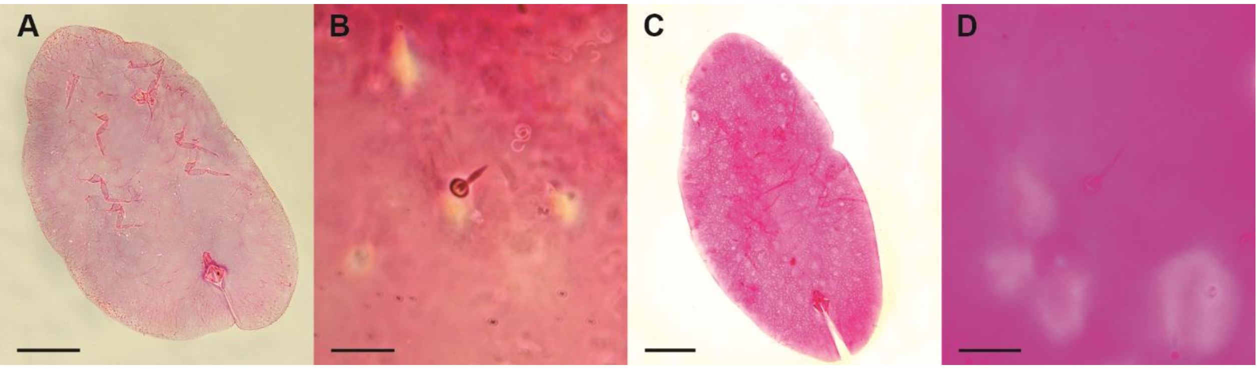

Figure S3. Microphotography of *Coccus hesperidum*, A: Body, B: Dorsal seta; *Coccus longulus*, C: Body, D: Dorsal seta. Scale bars: 10 µm (B, D), 500 µm (A, C).

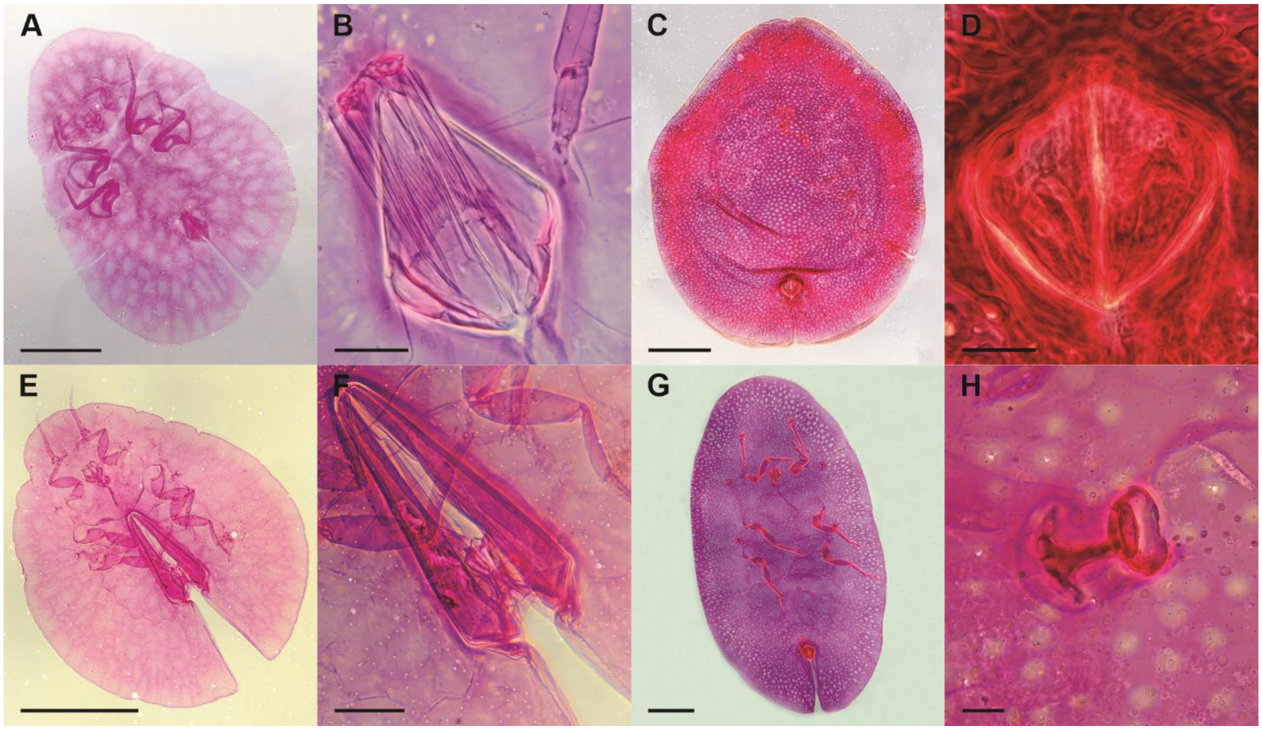

Figure S4. Microphotography of *Kilifia acuminata*, A: Body, B: Anal plates; *Parasaissetia nigra*, C: Body, D: Reticulation pattern, anal disc without discal seta; *Protopulvinaria pyriformis*, E: Body, F: Anal plates; *Pulvinaria psidii*, G: Body, H: Oval sclerotization around spiracles. Scale bars: 50 µm (B, D), 100 µm (F, H), 500 µm (A, C, E, G).

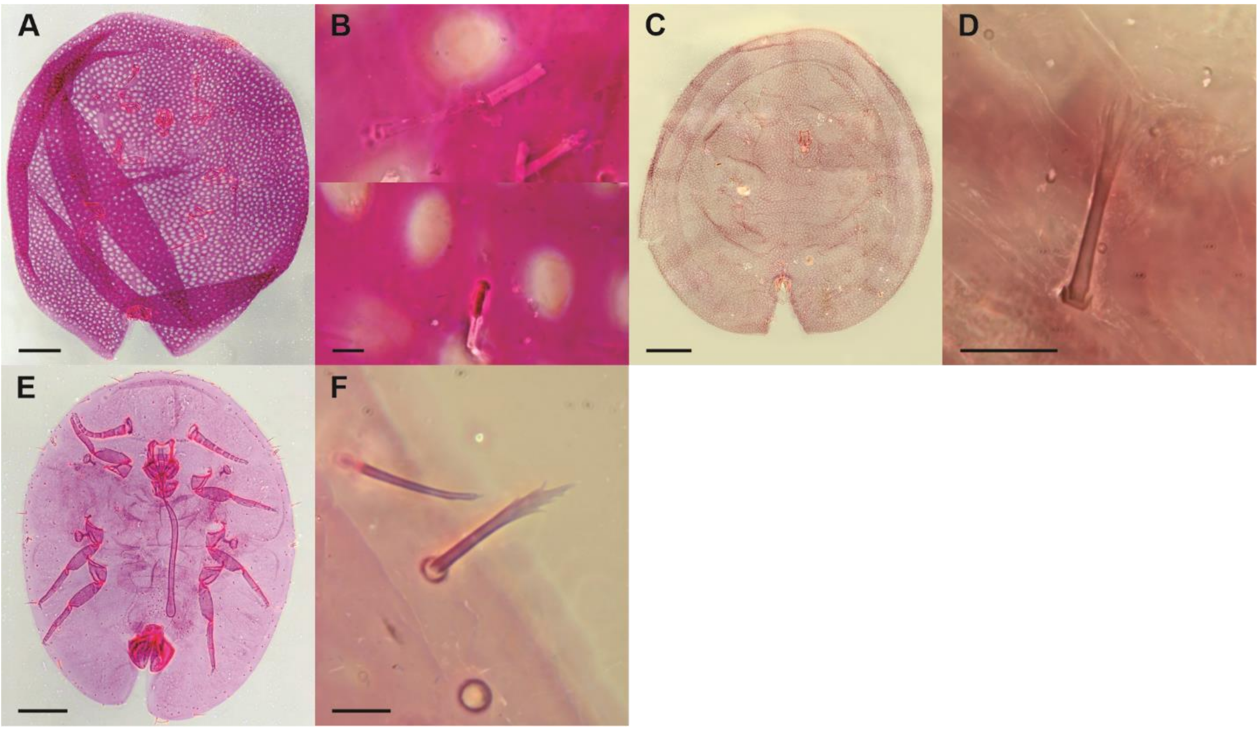

Figure S5. Microphotography of *Saissetia coffeae*, A: Body, B: Two types of submarginal ducts; *Saissetia neglecta*, C: Body, D: Marginal seta strongly fringed; *Saissetia oleae*, E: Body, F: Anterior marginal seta. Scale bars: 10 µm (B, F), 20 µm (D), 200 µm (E), 500 µm (A, C).

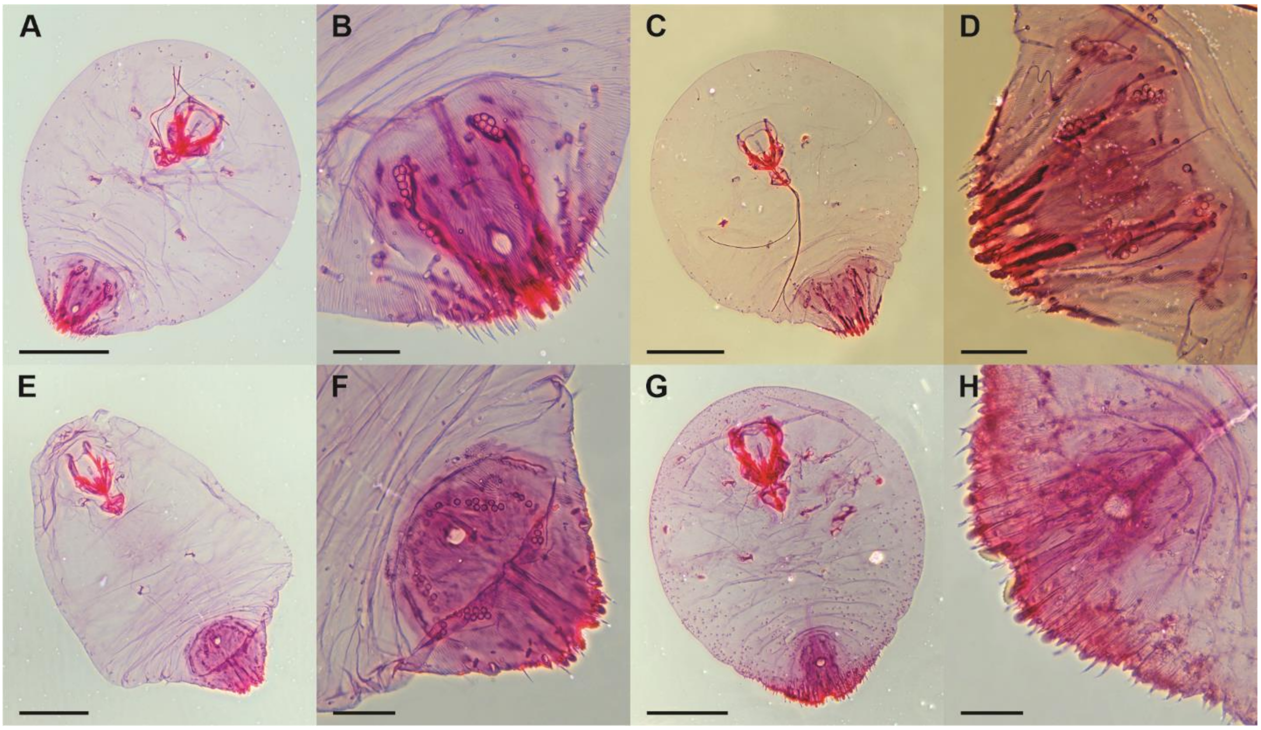

Figure S6. Microphotography of *Aspidiotus nerii*, A: Body, B: Pygidium; *Chrysomphalus dictyospermi*, C: Body, D: Pygidium; *Fiorinia fioriniae*, E: Body, F: Pygidium; *Furchadaspis zamiae*, G: Body, H: Pygidium. Scale bars: 50 µm (B, D, F, H), 200 µm (A, C, G), 500 µm (E).

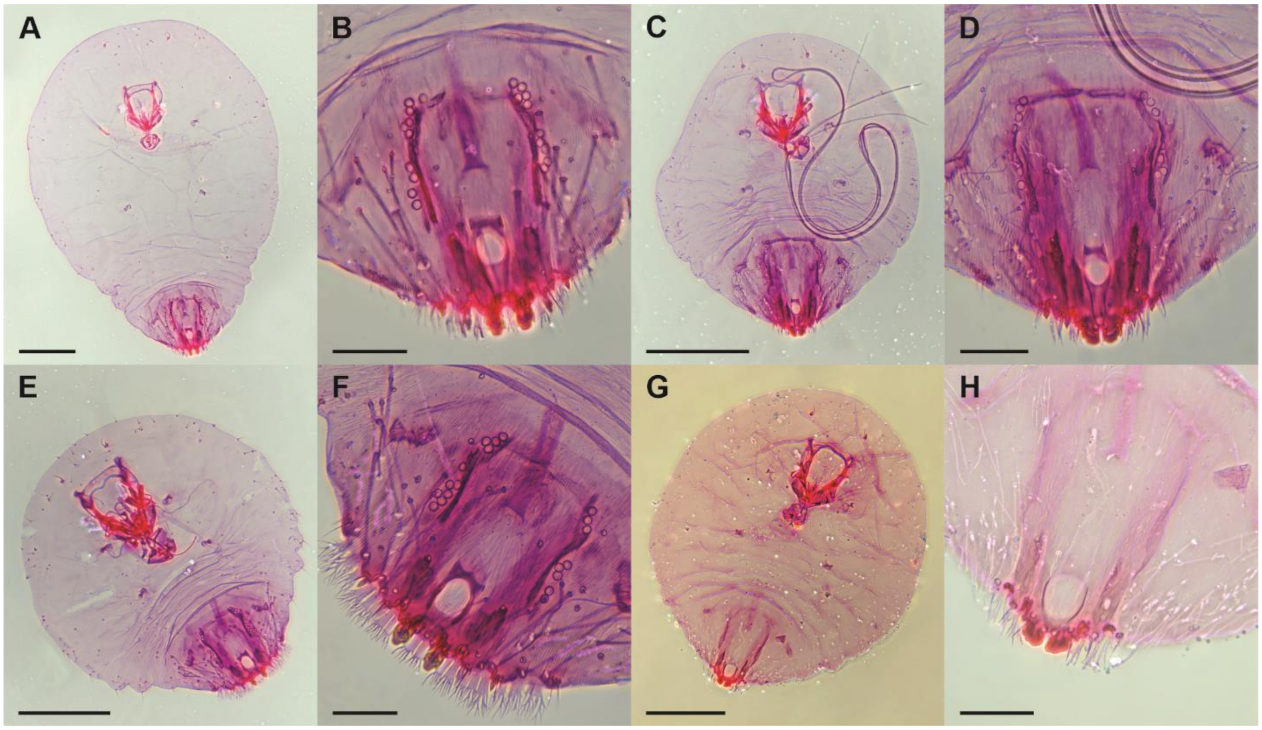

Figure S7. Microphotography of *Hemiberlesia cyanophylli*, A: Body, B: Pygidium; *Hemiberlesia lataniae*, C: Body, D: Pygidium; *Hemiberlesia palmae*, E: Body, F: Pygidium; *Hemiberlesia rapax*, G: Body, H: Pygidium. Scale bars: 50 µm (B, D, F, H), 200 µm (E, C, G), 500 µm (A).

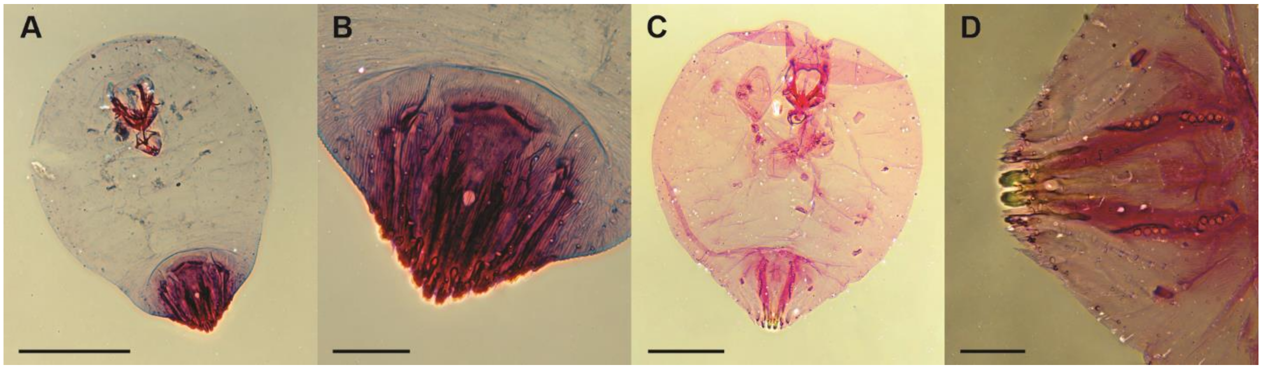

Figure S8. Microphotography of *Melanaspis* sp., A: Body, B: Pygidium; *Oceanaspidiotus spinosus*, C: Body, D: Pygidium. Scale bars: 50 µm (B, D), 200 µm (A, C).

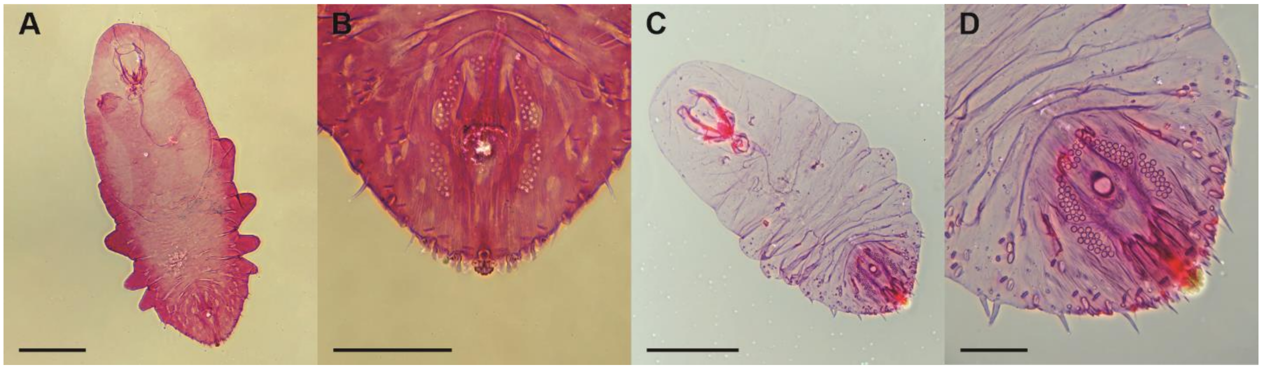

Figure S9. Microphotography of *Pinnaspis aspidistrae*, A: Body, B: Pygidium; *Pinnaspis strachani*, C: Body, D: Pygidium. Scale bars: 50 µm (B), 100 µm (D), 200 µm (A, C).

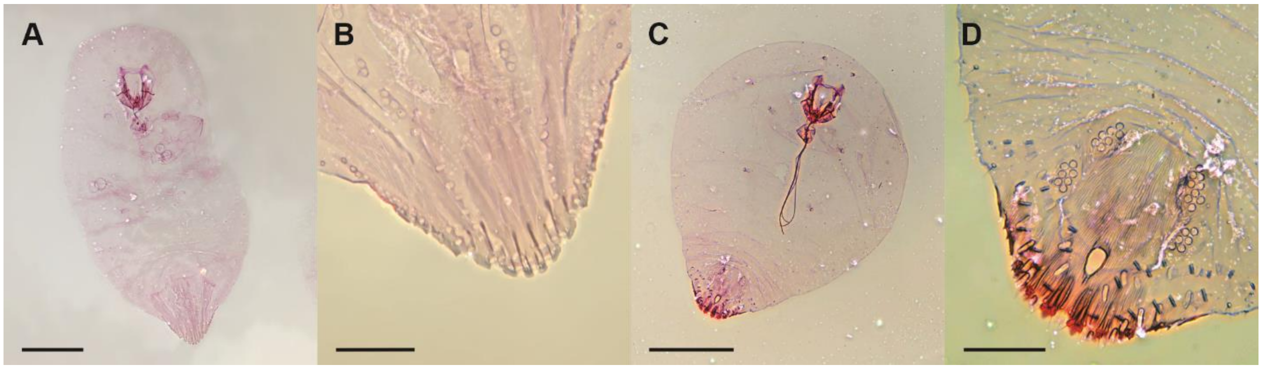

Figure S10. Microphotography of *Pseudischnaspis bowreyi*, A: Body, B: Pygidium; *Pseudoparlatoria parlatorioides*, C: Body, D: Pygidium. Scale bars: 50 µm (B, D), 200 µm (A, C).

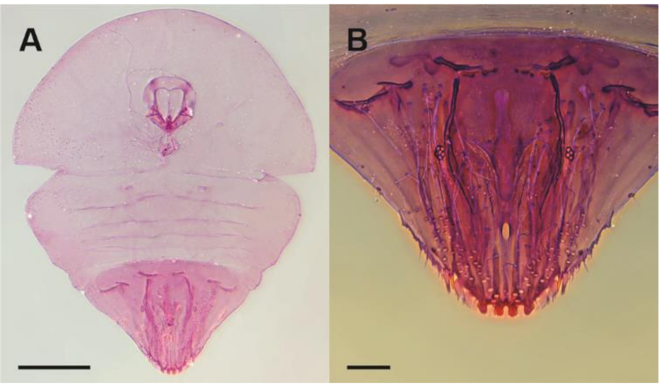

Figure S11. Microphotography of *Selenaspidus articulatus*, A: Body, B: Pygidium. Scale bars: 50 µm (B), 200 µm (A).

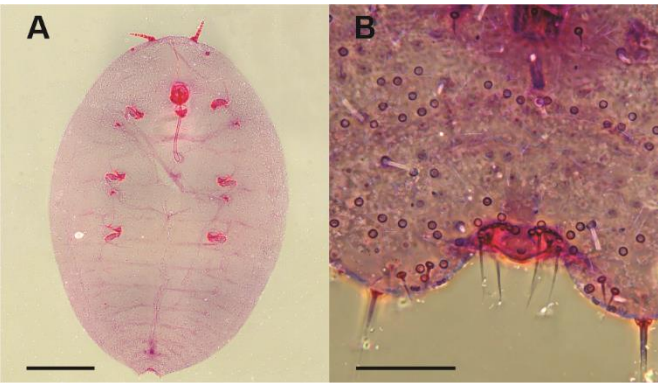

Figure S12. Microphotography of *Ovaticoccus peruvianus*, A: Body, B: Setae on anal disc. Scale bars: 50 µm (B), 500 µm (A).

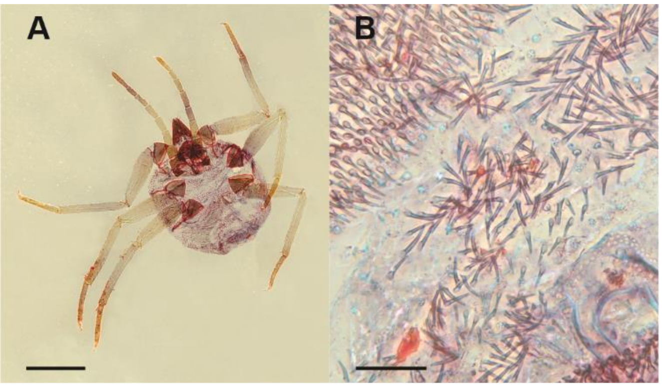

Figure S13. Microphotography of *Praelongorthezia olivicola*, A: Body, B: Pores inside ovisac band. Scale bars: 100 µm (B), 500 µm (A).

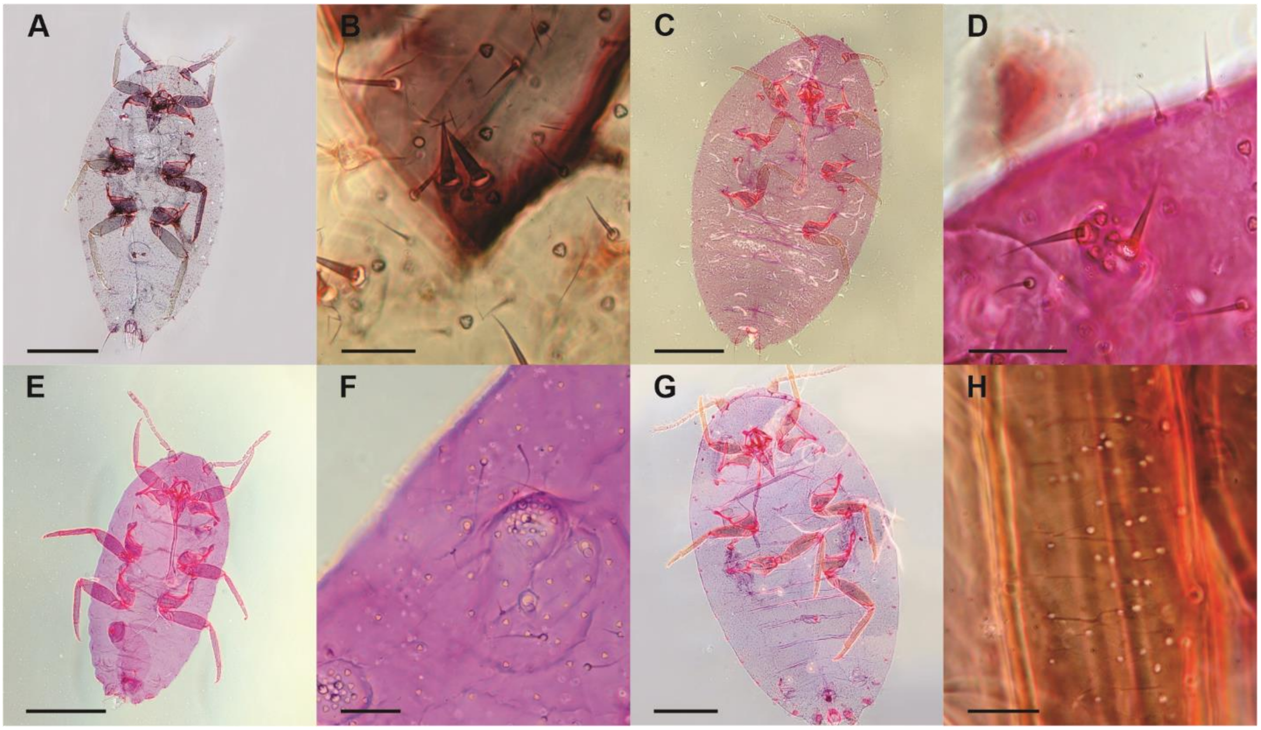

Figure S14. Microphotography of *Planococcus citri*, A: Body, B: Cerari with conic setae; *Planococcus ficus*, C: Body, D: Cerari with large setae; *Pseudococcus longispinus*, E: Body, F: Three oral ducts arranged beside cerarii; *Pseudococcus viburni*, G: Body, H: Translucid pores on tibia. Scale bars: 20 µm (B, D, H), 50 µm (F), 500 µm (A, C, E, G).
